# Bioactivity-guided analysis of *Moringa olifera* fractionated extracts for potential medical application

**DOI:** 10.1038/s41598-026-42314-4

**Published:** 2026-03-24

**Authors:** Nancy G. Banoub, Masarra M. Sakr, Mahmoud M. Tawfick, Shaimaa Fayez, Mohamed M. S. Farag, Khaled M. Aboshanab

**Affiliations:** 1 Department of Microbiology and Immunology, Faculty of Pharmacy, Heliopolis University, Cairo, 11785 Egypt; 2 Department of Microbiology and Immunology, Faculty of Pharmacy, Ain Shams University, Cairo, 11566 Egypt; 3 Department of Microbiology and Immunology, Faculty of Pharmacy (For Boys), Al-Azhar University, Cairo, 11751 Egypt; 4 Department of Pharmacognosy, Faculty of Pharmacy, Ain Shams University, Cairo, 11566 Egypt; 5 Department of Botany and Microbiology, Faculty of Science, Al-Azhar University, Cairo, 11884 Egypt; 6 Regional Mycology and Biotechnology Center, Al-Azhar University, Cairo, 11885, Egypt

**Keywords:** *Moringa oleifera*, Fractionation, Cytotoxicity, Antiviral activity, Expression analysis, Apoptosis, Biochemistry, Biotechnology, Cancer, Drug discovery, Plant sciences

## Abstract

*Moringa oleifera,* a nutrient-rich medicinal plant, has garnered growing scientific interest due to its diverse pharmacological properties. In this study, spectroscopic analysis, coupled with cytotoxic, antiviral, and antioxidant properties testing, was followed by gene expression analysis of various fractionated *Moringa oleifera* extracts to assess their potential biomedical applications. Ethanolic leaf extract was fractionated using solvents with increasing polarity (hexane, methylene chloride, ethyl acetate, and butanol). Cytotoxicity was tested against multiple cancer cell lines and normal cells, while antiviral activity was assessed against HAV ( Hepatitis A virus) and HSV-1/2 (Herpes simplex virus type 1/type 2). Antioxidant capacity was evaluated using DPPH assays. Gene expression of *BAX* and *BCL*-2 was analyzed by qRT-PCR to understand apoptotic pathways. Chemical profiling was performed using LC–MS/MS and GC–MS. The hexane fraction showed potent cytotoxicity against cancer cell lines MCF-7 (breast carcinoma) and HepG-2 (hepatocellular carcinoma), with low toxicity to normal PBMCs (normal human primary peripheral blood mononuclear cells). The butanol fraction displayed strong antiviral activity, particularly against HAV and HSV strains, while the ethyl acetate fraction exhibited the strongest antioxidant activity. The pro-apoptotic *BAX* gene was upregulated while the anti-apoptotic *BCL-2* gene was downregulated, according to gene expression analysis. Metabolomic profiling identified over 60 bioactive compounds, including phenolic acids, flavonoids, and terpenoids, which were consistent with the observed biological activities. These findings characterize *Moringa oleifera* as a rich source of multifunctional bioactive compounds and provide a preliminary in vitro foundation for its potential application in selective anticancer and antiviral therapies.

**Supplementary Information:**

The online version contains supplementary material available at 10.1038/s41598-026-42314-4.

## Introduction

Plants have long been considered effective medicinal agents due to their lower toxicity and fewer adverse effects than synthetic medications. Furthermore, phytochemical substances present in plants support a variety of complex cellular pathways^1,2^. *Moringa oleifera*, the “miracle tree”, is found across tropical and subtropical regions worldwide, even though Afghanistan, Bangladesh, India, and Pakistan are thought to be its original locations^3^. The *Moringa* family includes 13 species*,* of which *Moringa oleifera* is well known for its many purposes in medicine, nutrition, fertilizer, and biogas production^3^. According to reports, *Moringa oleifera* possesses several biological functions, including anti-inflammatory, anti-diabetic, hypotensive, and gastric ulcer-preventive effects^4^. It has also been demonstrated to control thyroid hormone levels and improve liver and kidney function**.** Additionally, *Moringa oleifera* leaves protect against liver damage, oxidative stress, cancer, and viral infections^5^. Fractions from the *Moringa oleifera* extract have been produced using various solvents, including ethanol, ethyl acetate, hexane, butanol, and methanol. These fractions have been observed to exhibit various properties, including antitumour, antioxidant, antiviral, immunomodulatory, antimicrobial, and radioprotective effects^6^. *Moringa oleifera* is known for its numerous bioactive compounds. The most often utilized components of the plant, the leaves, are notably rich in flavonoids, phenolic acids, vitamins, polyphenols, carotenoids, alkaloids, glucosinolates, isothiocyanates, tannins, and saponins, which are thought to be responsible for its diverse biological characteristics. Several in vitro and in vivo studies have confirmed these pharmacological properties^7^.

*Moringa oleifera* has shown antiviral properties against influenza and herpes simplex viruses by inhibiting viral replication and modulating immune responses^8^. It also exhibits anticancer effects, with research indicating that *Moringa* extracts can reduce tumor growth and stimulate apoptosis in numerous cancer cell lines^9^. Additionally, *Moringa oleifera* is a strong antioxidant that scavenges free radicals and reduces oxidative stress, thereby helping prevent chronic diseases^9^. Moreover, *Moringa oleifera* possesses immunomodulatory effects, enhancing immune function by promoting cytokine synthesis and strengthening the body’s defence mechanisms against infections and diseases^5^. Although previous studies have explored solvent-based extractions or examined the modulation of apoptotic markers such as *BAX* and *BCL-2* in response to *Moringa oleifera* extracts, these investigations were often limited in scope, focusing on single bioactivities, fewer cell lines, or lacking detailed chemical characterization. The present study bridges these gaps by regarding how systematic fractionation using solvents of varying polarities can enrich specific metabolites and, consequently, modulate their biological efficacy. The novelty of this study lies in its comparative, bioactivity-guided approach. Rather than evaluating a single extract, we systematically fractionated the crude ethanolic extract using a sequence of increasing polarity: hexane, methylene chloride, ethyl acetate, and n-butanol. This allows for a precise mapping of the chemical constituents—identified via GC–MS and LC–MS/MS—against their specific cytotoxic and antiviral potencies. Therefore, this study aimed to isolate, extract, and fractionate ethanolic *Moringa oleifera* extract to identify bioactive compounds with potential in vitro biological activity. relevance. *Moringa oleifera* extract and various solvent fractions were examined for the antioxidant, cytotoxic, and antiviral properties. Furthermore, the antiviral activity of the most active fractions was assessed against specific immune cells. The study also assessed the expression levels of key apoptotic genes and identified and characterized the most active metabolites. The comparative evaluation of multiple solvent fractions derived from a single ethanolic extract, the distinct bioactivity profiles (cytotoxic, antiviral, antioxidant) of each fraction, and the association of these activities with dominant chemical classes, as revealed by LC–MS/MS and GC–MS analysis.

## Materials and methods

### Collection and processing of *Moringa oleifera* leaves

The *Moringa oleifera* plant material used in this study was collected from authenticated trees cultivated at the SEKEM Farm (Belbis, Sharkia Governorate, Egypt) in April 2023, Cairo, Egypt. The sample was received with an official Quality Control label documenting the sample name, number, and collection date, ensuring full traceability. The collection of plant material was established in compliance with the national guidelines. To further confirm the plant identity, the plant was kindly authenticated by Mrs Trease Labib, Senior Botanist at Mazhar Botanical Garden, Cairo, Egypt. A voucher specimen no. (PHG-P-MO-562) was deposited in the Herbarium of the Department of Pharmacognosy, Faculty of Pharmacy, Ain Shams University, Cairo, Egypt. *Moringa oleifera* leaves were extracted as previously described by Krishnamurthy et al.^10^. Approximately 900 g of fresh *Moringa oleifera* leaves were carefully washed under running tap water to get rid of dust and other debris. The leaves were then allowed to air-dry for two days at room temperature. The dried plant parts were coarsely ground in a grinder, yielding 218 g of powdered leaves.

### Preparation of ethanolic *Moringa oleifera* extract and fractionation

The *Moringa oleifera* leaves powder was macerated with 1 L of 96% ethanol for 48 h at room temperature. The resultant mixture was filtered through filter paper, the filtrate was collected and evaporated under vacuum at 40 °C using a rotary evaporator, yielding 18 g of a dark green, oily extract. The extract was stored at 10 °C for further use^10^. The ethanolic extract was then subjected to sequential liquid–liquid fractionation using a series of solvents with increasing polarity: hexane, methylene chloride, ethyl acetate, and n-butanol. For each stage, the extract was partitioned with the respective solvent in a 1:1 (v/v) ratio, and the process was repeated three times to ensure exhaustive extraction of the bioactive constituents. Each resulting solvent fraction was evaporated under reduced pressure at 40°C and subsequently freeze-dried to obtain stable, dry residues. A rotary evaporator was used to dry the hexane, ethyl acetate, methylene chloride, and butanol fractions under vacuum at 40 °C before they were freeze-dried. The ethanolic *Moringa oleifera* extract and its solvent fractions were then utilized for experimental studies and referred to as tested samples^11^.

### Cell lines and cell culture techniques

Normal human primary peripheral blood mononuclear cells (PBMCs) (ATCC® PCS-800011™) and normal animal Vero cells derived from the kidney of African green monkey (ATCC® CCL- 81™) were purchased from Vascera, Cairo, Egypt. The cancer cell lines A-549 (lung carcinoma), Panc-1 (pancreatic carcinoma), Caco-2 (intestinal carcinoma), HELA (cervical carcinoma), HepG-2 (human hepatocellular carcinoma), and MCF-7 (human breast carcinoma) were also acquired from Vascera, Cairo, Egypt. Dulbecco’s modified Eagle’s medium (DMEM, Invitrogen, Carlsbad, CA, USA) containing 10% heat-inactivated fetal bovine serum (Hyclone, Marlborough, MA, USA), 1% L-glutamine (Sigma-Aldrish St. Louis, MO, USA), HEPES buffer and 50 µg/mL gentamicin (Grand Island, NY, USA) was used to maintain the MCF-7, HepG-2, A-549, Panc-1, Caco-2, and HELA cancer cell lines^12^. The Roswell Park Memorial Institute (RPMI) medium (Lonza, Germany) supplemented with 2% serum was used to cultivate *Vero* cells and PBMCs. In the assays performed in the current study, the cells were incubated at 37 °C in a humidified atmosphere with 5% CO_2_, and the cultures were subcultured twice weekly.

### Cytotoxicity evaluation

The cytotoxicity of the tested samples was evaluated against PBMCs and *Vero* cells using the 3-(4,5-dimethylthiazol-2-yl)-2,5-diphenyl tetrazolium bromide (MTT) assay, as described by El Sayed et al.^13^, where absorbance was finally measured at 570 nm by a microplate reader (SunRise, TECAN, Inc., USA). Untreated cells were used as negative controls, while cells treated with cisplatin (Sigma-Aldrich, Darmstadt, Germany) served as positive controls^13^. To ensure reproducibility, all experiments were performed in triplicate. The cytotoxic effects of the tested samples were evaluated against several cancer cell lines, including MCF-7, HepG-2, Caco-2, A-549, and HeLa. Cytotoxicity was assessed using the crystal violet staining assay, as previously described by Feoktistova et al.^14^. Cells were incubated with the tested samples for 24 h at 37 °C in a humidified atmosphere containing 5% CO₂^14^. Cell viability was then measured by a colourimetric method, with absorbance of each well recorded at 570 nm using a microplate reader (TECAN, Inc.). Vinblastine sulfate-treated cells were used as positive controls, whereas untreated cells served as negative controls^14^. All experiments were performed in triplicate to ensure reproducibility.

### Evaluation of the antiviral activity

#### Virus and cell culture

The antiviral activity of the tested samples was evaluated against Hepatitis A virus (HAV HM175 strain), Herpes simplex virus type 1 (HSV-1, GHSV-UL46 strain), and Herpes simplex virus type 2 (HSV). The viral strains were purchased from Nawah Scientific (Mokattam, Egypt) and propagated at the Regional Center for Mycology and Biotechnology (RCMB) et al.-Azhar University, Cairo, Egypt.

#### Virus propagation

Vero cells were seeded in cell culture flasks one day before the virus strain was propagated and assayed in confluent Vero cells^14^. Using the Spearman-Karber method, infectious viruses were quantified by calculating the 50% tissue culture infectious dose (TCID50) in eight wells per dilution, with 20 µL of inoculum per well^15^.

#### Antiviral activity evaluation

A cytopathic effect (CPE) inhibition assay, which measures the ability of the tested sample to inhibit virus-induced cytopathic effects in susceptible mammalian cells. A cytopathic effect inhibition assay was used to evaluate the antiviral activity of the tested samples. The assay was performed using the MTT method^16^. For each tested sample, Vero cell monolayers were added to 96-well plates at a density of 2 × 10^5^ cells/well and incubated for 24 h at 37 °C in a humidified atmosphere containing 5% CO₂. The monolayers were washed, and a standardized viral titer of 10^4^ TCID50 per well was used to challenge the cells (HAV, HSV-1, or HSV-2). The infected cells were then overlaid with RPMI medium containing 2% serum (100 µL) and various concentrations (0—1000 µg/mL) of the tested sample. The plates were incubated at 37 °C for 48 h. Infection controls (virus-infected cells without treatment) and untreated Vero cell controls (uninfected and untreated) were included as negative controls, while acyclovir was used as a positive control. After the incubation period, the MTT assay was used to assess cell viability^16^. The antiviral activity was calculated based on the ability of the tested sample to inhibit viral cytopathic effects. All experiments were performed in triplicate, and GraphPad Prism (GraphPad Software, San Diego, CA, USA) was used to determine the 50% inhibitory concentration (IC₅₀) and 50% cytotoxic concentration (CC₅₀). As described by Dogan et al.^17^, the selectivity index (SI) was calculated using the following formula: selectivity index (SI) = CC50/IC50^17^

### Antioxidant activity assay

#### DPPH radical scavenging activity

To evaluate the antioxidant property of the tested samples, the 2,2-diphenyl1-1-picrylhydrazyl (DPPH) free radical scavenging assay was performed according to the method described by Lu et al*.*^18^. The examined samples were tested using a two-fold serial dilution ranging from 1000 µg/mL down to 0 µg/mL. Similarly, ascorbic acid, used as a positive control, was subjected to the same two-fold serial dilution to accurately determine its IC₅₀ value. The negative control consisted of the DPPH radical solution (0.004% w/v in methanol) without any antioxidant. All experiments were performed in triplicate. The % inhibition (PI) of the DPPH radical was calculated as previously reported^19^. The IC50, or sample concentration needed to block half of the free radicals, was calculated^12^.

#### Quantitative real-time PCR (qRT-PCR)

The qRT-PCR analysis was conducted to investigate the effect of ethanolic extract, ethyl acetate fraction, and hexane fractions on the expression of *BCL-2* and *BAX* apoptotic genes in cancer cells (liver carcinoma, breast carcinoma, and pancreatic carcinoma cells)^20^. RNA extraction was performed according to the manufacturer’s instructions for the RNeasy Mini Kit from Qiagen (Catalogue no.74104). The PCR reaction was prepared using the Quantitect SYBR Green PCR Kit, as directed by the manufacturer, with a total reaction volume of 25 μL. Table 1 summarises the primer sequences used in this investigation.

**Table 1:** PCR oligonucleotide primers sequences.

Gene	Primer sequence (5’-3’)	Expected Amplicon Sizes	Reference
GAPDH (Glyceraldehyde-3-Phosphate-Dehydrogenase)	CTCTGATTTGGTCGTATTGGG	150 bp	^33^
	TGGAAGATGGTGATGGGATT		
BAX (BCL-2-Associated x gene)	GGTTGTCGCCCTTTTCTA	116bp	^47^
	CGGAGGAAGTCCAATGTC		
BCL2 (B-Cell Lymphoma 2)	GATGTGATGCCTCTGCGAAG	128bp	
	CATGCTGATGTCTCTGGAATCT		

#### Cycling conditions for SYBR Green real-time PCR

After 30 min of reverse transcription at 50 °C, 94 °C was used for 15 min of initial denaturation. There were 40 cycles in the amplification cycle: 15 s of denaturation at 94 °C, 30 s of annealing (58 °C for GAPDH, 50 °C for *BAX*, and 64 °C for *BCL-2*), and 30 s of extension at 72 °C^20^. A dissociation curve analysis was performed in sequential steps: secondary denaturation at 94 °C for 1 min, annealing at the gene-specific temperature for 1 min, and final denaturation at 94 °C for 1 min.

#### Analysis of the SYBR Green RT-PCR

Amplification curves and cycle threshold (CT) values were calculated using the Stratagene MX3005P software. Gene expression levels were analyzed using the ^ΔΔ^CT method^20^. The relative fold change in gene expression was calculated using the formula 2^(-^ΔΔ^CT), with GAPDH serving as a housekeeping gene for normalization. All experiments were performed in triplicate, and the fold change was calculated using the average CT values. The ΔΔCT values were calculated as previously described^20^.

#### LC–MS/MS analysis of the ethanolic *moringa oleifera* extract, butanol fraction, and ethyl acetate fraction

The analysis of the ethanolic *moringa oleifera* extract, butanol fraction, and ethyl acetate fraction was performed using LC–ESI–MS/MS with a UPLC apparatus with a reversed-phase column ( ACQUITY UPLC BEH C18 column, 1.7 µm particle size, 2.1 × 50 mm column) coupled to a Xevo TQD triple quadrupole system (Waters Corporation, Milford, MA01757, USA) with an electroscopy ESI source (ESI voltage = 3.0 kV; nitrogen as sheath gas; capillary temperature 440 °C)^21^. Chromatographic separation was performed at a flow rate of 0.2 mL/min using gradient elution (A: H2O containing 0.1% formic acid, B: Acetonitrile containing 0.1% formic acid). Both positive and negative modes of mass spectra were acquired (scan range: m/z 100–1000) at a collision-induced dissociation (CID) energy of 20 eV^22^. The spectra were analyzed using the MassLynx 4.1 software^23^.

#### Gas chromatography/mass spectroscopy (GC–MS) analysis

Hexane fraction of *Moringa oleifera* extract was analyzed using a Shimadzu GC–MS-QP 2010 (Kyoto, Japan) with a Rxi-1MS fused bonded column ( 30m × 0.25 mm i.d., 0.25 × 0.25 μm film thickness, Restek, USA) fitted with a split-splittles injector^24^. The diluted samples (1% v/v) were injected in a split mode with a split ratio of 1:30. This ratio was specifically chosen to prevent column overloading and to ensure sharp, well-resolved peaks for both major and minor constituents in the extract. After 3 min at the initial column temperature of 50 °C, it was programmed to reach 300 °C at 5 °C/min; it remained at 300 °C for 10 min (isothermal). The injector’s temperature was set at 280 °C. 1.37mL/min was maintained as the helium carrier gas flow rate. The following parameters were used to record mass spectra: ion source temperature (220 °C), filament emission current (60 mA), and ionization voltage (70 eV), and the diluted samples (1% v/v) were injected as previously described^25^.

### Statistical analysis

Unless otherwise noted, the mean ± standard deviation (SD) of at least three independent replicates is used to express descriptive results for all experiments. For statistical analysis, GraphPad Prism (GraphPad Software, San Diego, CA, USA) was used. Nonlinear regression analysis was used to determine the 50% inhibitory concentration (IC₅₀) values, which were used to evaluate cytotoxicity. The selectivity index (SI) was calculated from dose–response curves to evaluate antiviral activity. Fold changes were calculated using the ^ΔΔ^CT technique to evaluate gene expression changes. All experiments were conducted in triplicate to guarantee reproducibility, and the data were normalized to appropriate controls as described in the respective sections.

## Results

### Cytotoxic activity against PBMCs and Vero cells

Cytotoxic effects of the tested samples were evaluated against PBMCs and Vero cells. The IC50 values of PBMCs were determined as follows: ethanolic *Moringa oleifera* extract (624.31 ± 29.07 µg/mL), ethyl acetate fraction (77.76 ± 2.1 µg/mL), butanol fraction (126.92 ± 2.91 µg/mL), methylene chloride fraction (114.59 ± 3.47 µg/mL), and hexane fraction (93.27 ± 1.27 µg/mL). The IC50 values of Vero cells were determined as follows: ethanolic *Moringa oleifera* extract (647.4 ± 9.31µg/mL), ethyl acetate fraction (155.63 ± 2 µg/mL), butanol fraction (483.1 ± 9.2 µg/mL), methylene chloride fraction (327.96 ± 2.93 µg/mL), and hexane fraction (224.42 ± 5.18µg/mL). Cisplatin, utilized as a positive control, exhibited an IC50 value of 164.08 ± 0.78 µg/mL. These results indicated that all tested samples exhibited low cytotoxicity toward PBMCs and Vero cells. Among the solvent fractions, the ethyl acetate fraction showed the highest cytotoxic activity; however, this value remains well above the NCI’s high-cytotoxicity threshold (IC50 < 20 µg/ml), indicating a safe profile, while the ethanolic *Moringa oleifera* extract exhibited the lowest cytotoxicity.

### Cytotoxic activity against cancer cell lines

**Fig. 1:**
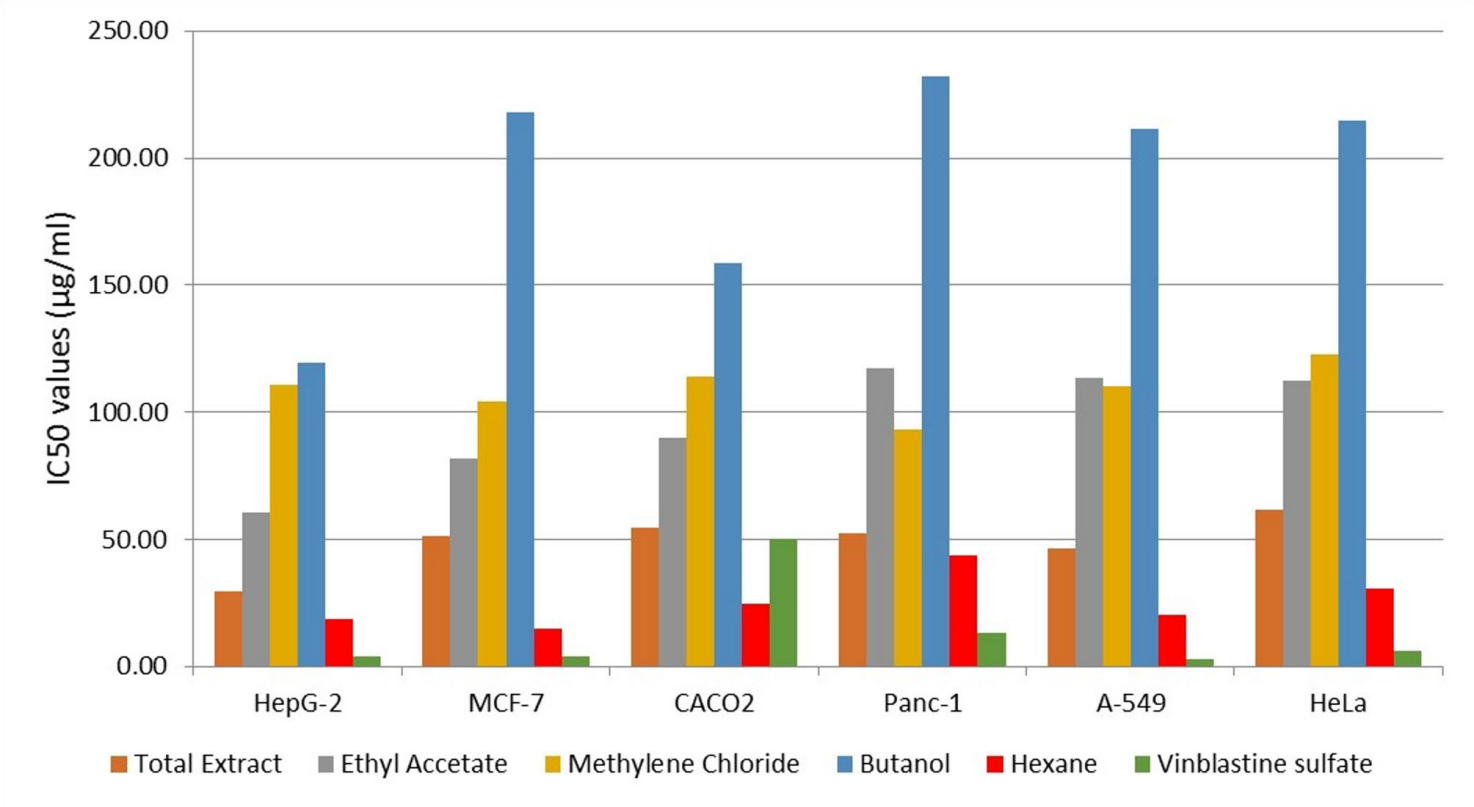
Anticancer activities of the crude ethanolic *Moringa oleifera* extract and its fractions.

As depicted in the dose–response curves (Figure S1), and the comparative viability bar charts (Fig. 1), all *Moringa oleifera* fractions exhibited a concentration-dependent reduction in cell viability. Notably, the hexane fraction and the total ethanolic extract demonstrated a remarkable safety profile, with the highest IC₅₀ values in normal PBMCs, resulting in superior Selectivity Index (SI) values exceeding 10.0 across all tested cancer lines (Table 2). The hexane fraction also maintained a favorable selectivity window (SI > 2), with its highest selectivity observed against breast cancer cells (SI = 6.30).In contrast, the ethyl acetate, methylene chloride, and butanol fractions showed moderate to weak cytotoxicity, with SI values generally near or below 2.0, indicating lower specificity for cancer cells. While the reference drug, Vinblastine Sulfate, displayed the highest absolute potency IC₅₀ for most cancer cell lines, its selectivity against several lines was comparable to or lower than that of the hexane fraction. These findings, supported by thedose-response curves in Figure S1, highlight the hexane fraction as the most selective and potent bioactive fraction derived from *Moringa oleifera* leaves.

**Table 2:** Cytotoxic activity (IC50) and selectivity indices (SI) of *Moringa oleifera* extracts and fractions against cancer cell lines compared to normal human PBMCs.

Sample code	PBMC IC50 (µg/mL)	Cancer cell line	IC50 (µg/mL)	SI
Ethanolic extract	624.31 ± 29.07	MCF-7	51.53 ± 2.05	12.1
		HepG-2	29.68 ± 0.63	21
		CACO2	54.82 ± 0.78	11.4
		Panc-1	52.57 ± 2.19	11.9
		A-549	46.46 ± 1.79	13.4
		HeLa	61.90 ± 0.53	10.1
Ethyl Acetate fraction	77.76 ± 2.1	MCF-7	81.93 ± 3.98	0.9
		HepG-2	60.71 ± 0.42	1.3
		CACO2	89.88 ± 2.28	0.8
		Panc-1	117.13 ± 2.52	0.6
		A-549	113.51 ± 4.09	0.7
		HeLa	112.32 ± 1.89	0.7
Methylene chloride fraction	114.59 ± 3.47 µg/mL	MCF-7	104.11 ± 1.91	1.1
		HepG-2	110.87 ± 1.91	1
		CACO2	113.80 ± 1.73	1
		Panc-1	93.00 ± 1.27	1.2
		A-549	110.13 ± 4.77	1
		HeLa	122.39 ± 0.97	0.9
Butanol extract	126.92 ± 2.91	MCF-7	218.11 ± 2.08	0.6
		HepG-2	119.22 ± 2.12	1.1
		CACO2	158.38 ± 5.90	0.8
		Panc-1	231.92 ± 4.76	0.5
		A-549	211.66 ± 10.29	0.6
		HeLa	214.67 ± 4.18	0.6
Hexane extract	93.27 ± 1.27 µg/mL	MCF-7	14.81 ± 0.32	6.3
		HepG-2	18.34 ± 0.76	5.1
		CACO2	24.78 ± 1.04	3.8
		Panc-1	43.82 ± 2.41	2.1
		A-549	20.48 ± 0.10	4.6
		HeLa	30.44 ± 0.49	3.1
Vinblastine Sulfate	of 164.08 ± 0.78 µg/mL	MCF-7	3.78 ± 0.54	4.89
		HepG-2	3.68 ± 0.17	5.02
		CACO2	50.3 ± 6.10	0.36
		Panc-1	13.27 ± 0.94	1.39
		A-549	2.84 ± 0.32	6.51
		HeLa	6.14 ± 0.92	3.01

Data are expressed as Mean ± SD (n = 3). SI (Selectivity Index) = IC50of normal PBMCs / IC50 of cancer cells. SI > 2 indicates high selective toxicity toward cancer cell.

### Cytopathic effect of the tested samples against Viral Cell Lines

Results displayed in Table 3 revealed promising replication-inhibitory activity and showed that the butanol fraction demonstrated the most potent antiviral activity against all three viruses, with a high SI value confirming that the viral inhibition occurs at concentrations well below the cytotoxic threshold. These values indicate a favorable safety margin for this initial in vitro screening. The ethyl acetate fraction showed significant antiviral activity, particularly against HSV-1 and HSV-2, with moderate SI values. The hexane fraction demonstrated moderate antiviral activity against HSV-1 and HSV-2 but was inactive against HAV. The ethanolic extract and methylene chloride fraction showed weak or no antiviral activity against all tested viruses. As expected, acyclovir, the positive control, exhibited the most effective antiviral activity with the highest SI.

**Table 3:** Antiviral activity of the tested samples.

Virus	Sample	IC50 (µg/mL)	CC50 (µg/mL)	SI
HAV	Ethanolic extract	Weak	270.33 ± 11.04	Inactive
	Ethyl Acetate fraction	51.23 ± 0.96	201.16 ± 4.38	3.923
	Methylene chloride fraction	Weak	419.04 ± 12.36	Inactive
	Butanol fraction	24.42 ± 1.09	176.2 ± 4.31	7.21
	Hexane fraction	Weak	161.98 ± 2.53	Inactive
	Acyclovir	14.75 ± 0.3	297.39 ± 3.47	20.16
HSV-1	Ethanolic extract	93.88 ± 2.85	270.33 ± 11.04	2.88
	Ethyl Acetate fraction	14.75 ± 0.33	201.16 ± 4.38	13.63
	Methylene chloride fraction	Weak	419.04 ± 12.36	Inactive
	Butanol fraction	13.23 ± 0.33	176.2 ± 4.31	13.32
	Hexane fraction	29.74 ± 0.41	161.98 ± 2.53	5.45
	Acyclovir	3.07 ± 0.15	138.54 ± 1.85	45.12
HSV-2	Ethanolic extract	Weak	270.33 ± 11.04	Inactive
	Ethyl Acetate fraction	25.41 ± 1.81	201.16 ± 4.38	7.19
	Methylene chloride fraction	Weak	419.04 ± 12.36	Inactive
	Butanol fraction	15.15 ± 0.28	176.2 ± 4.31	11.63
	Hexane fraction	47.37 ± 1.12	161.98 ± 2.53	3.41
	Acyclovir	6.95 ± 0.36	138.54 ± 1.85	19.93

CC50 is the half-maximal cytotoxic concentration; IC50 is the half-maximal inhibitory concentration; and SI is the selectivity index, calculated as CC50 divided by IC50.

### Antioxidant activity

Results in Table 4 show that the ethyl acetate fraction exhibited the most potent free radical scavenging activity among all the tested samples, indicating the presence of a highly effective antioxidant. The butanol fraction showed moderate antioxidant activity, while the ethanolic extract and hexane fraction showed weaker effects. The methylene chloride fraction exhibited the least antioxidant activity.

**Table 4:** The IC50 and standard deviation values of the tested samples in the DPPH.

Sample	IC50 (µg/mL) DPPH scavenging %
Ethanolic extract	109.55 ± 1.88
Ethyl Acetate fraction	14.66 ± 0.38
Methylene chloride fraction	459.39 ± 14.77
Butanol fraction	60.77 ± 0.75
Hexane fraction	229.28 ± 2.10
Ascorbic acid	10.21 ± 0.77

### Effect of ethanolic *Moringa oleifera* extract, ethyl acetate fraction and hexane fraction on the expression of *BCL-2 *and *BAX* genes in Cancer Cells

The effect of ethanolic *Moringa oleifera* extract, ethyl acetate fraction, and hexane fraction (1000 μg/mL) on the expression of the apoptosis-related genes *BCL-2* and *BAX* was evaluated in liver carcinoma, breast carcinoma, and pancreatic carcinoma cells after 24 h of treatment with the tested sample. Real-time PCR was used to analyze gene expression levels and was normalized to the reference gene GAPDH. Table 5 demonstrates a huge increase in *BAX* gene expression, accompanied by a huge reduction in *BCL*-2 expression compared to untreated cells. Representative qPCR amplification plots and melting curves for the target and reference genes are provided in the Supplementary Materials (Figure S2) to confirm the specificity and efficiency of the amplification process.

**Table 5:** Effect of ethanolic *Moringa oleifera* extract, ethyl acetate fraction, and hexane fraction on mRNA expression of apoptosis markers (*BCL-2* and *BAX*).

Sample	GAPDH	*BAX*		*BCL2*	
	CT	CT	Fold change	CT	Fold change
Liver carcinoma	20.43	22.95	–	20.68	–
Liver carcinoma + ethanolic extract	20.81	20.16	9.0005	22.04	0.5070
Liver carcinoma + ethyl acetate fraction	19.72	19.83	5.3147	20.29	0.8011
Liver carcinoma + hexane fraction	19.54	18.44	12.2950	21.95	0.2238
Breast carcinoma	19.25	21.89	–	20.14	–
Breast carcinoma + ethanolic extract	18.47	17.59	11.4716	20.87	0.3511
Breast carcinoma + ethyl acetate fraction	19.62	19.62	6.2333	21.22	0.6113
Breast carcinoma + hexane fraction	18.58	17.21	19.1113	23.56	0.0587
Pancreatic carcinoma	20.11	23.14	–	21.71	–
Pancreatic carcinoma + ethanolic extract	19.24	20.13	4.4076	21.68	0.5586
Pancreatic carcinoma + ethyl acetate fraction	20.18	22.10	2.1585	22.09	0.8066
Pancreatic carcinoma + hexane fraction	20.56	20.52	8.3977	23.27	0.4633

### LC–MS/MS analysis of the ethanolic *moringa oleifera* extract, butanol fraction, and ethyl acetate fraction

**Fig. 2:**
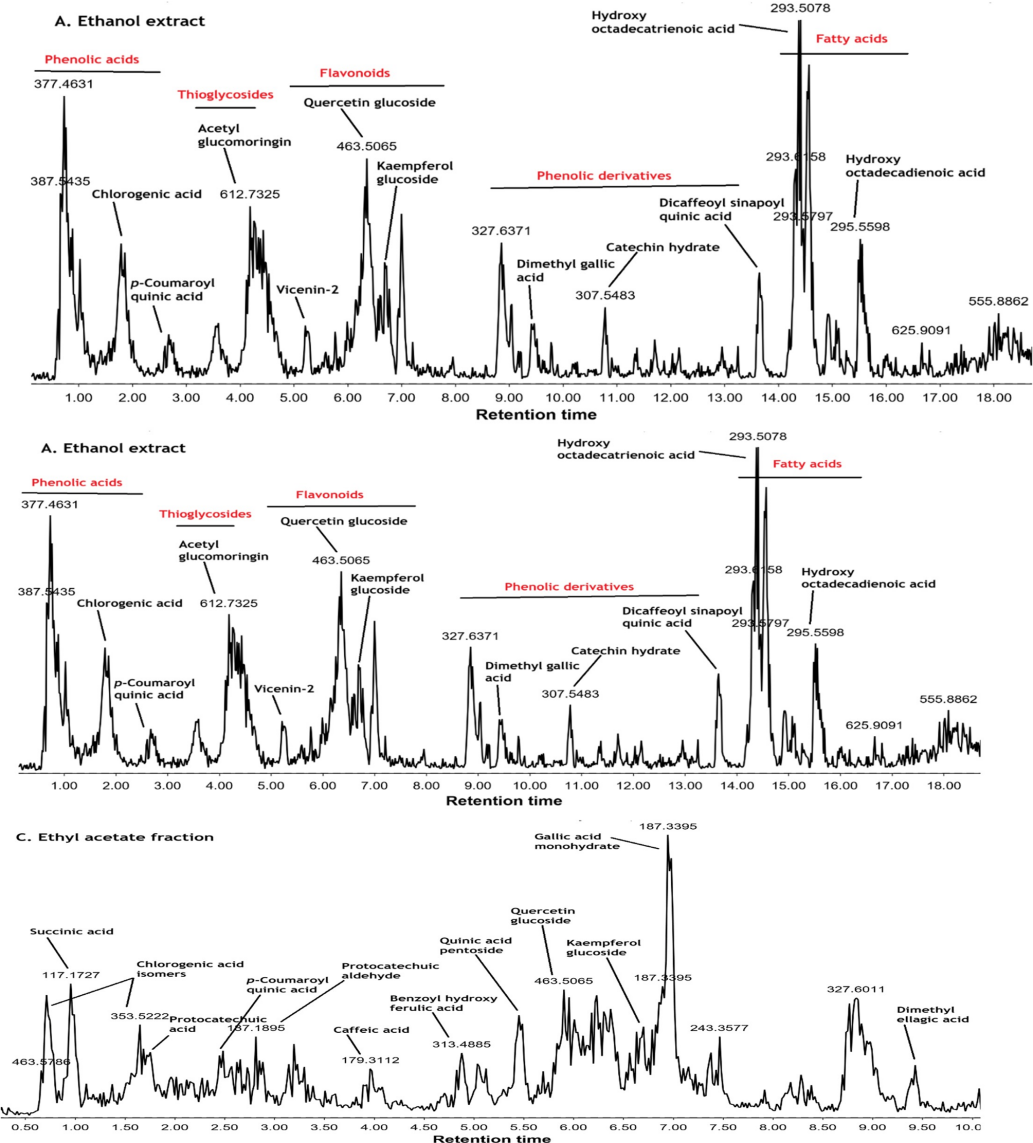
LC–MS chromatogram of the (**A**) ethanol, (**B**) butanol, and (**C**) ethyl acetate extracts of *Moringa oleifera* leaves in negative mode.

Qualitative analysis of the bioactive ethanolic *Moringa oleifera* extract butanol and ethyl acetate fractions, which displayed promising antimicrobial activities, using ESI–MS/MS, led to the putative identification of more than 20 metabolites belonging to flavonoids, phenolic acids, and fatty acids (Table 6). Despite the enrichment of *Moringa* seeds with glucosinolates (thioglycosides), only acetyl glucomoringin (peak 4) in the leaves was detected, with a deprotonated molecular ion peak at m/z 612 and a prominent fragment ion at m/z 97 (HSO4). Identification of compounds was based on analyzing their MS2 fragments and comparing them with those reported in the literature. Compared to the ethanolic extract (Fig. 2), the butanol fraction was enriched in some phenolic acids, such as p- p-coumaroyl quinic acids and chlorogenic, along with some flavonoids, specifically vicenin-2 and kaempferol acetyl glycoside (Fig. 2). On the other hand, thioglycosides, such as acetylglucosamine, were less intense and lacked fatty acids. The ethyl acetate fraction exhibited diversity in phenolic acids, including p- p-coumaroyl quinic acids, chlorogenic acid, protocatechuic acid, caffeic acid, and a ferulic acid derivative (Fig. 2). Some flavonoids, such as quercetin and kaempferol glucosides, were particularly enriched in the ethyl acetate fraction. The ethyl acetate fraction showed the absence of thioglycosides and fatty acids. The results of the chemical profiling revealed that phenolic acids and flavonoids are the primary phytochemical classes contributing to the pronounced antimicrobial activity observed in the fractions, rather than fatty acids or thioglycosides.

**Table 6:** LC–MS/MS-based identification of the main metabolites in *Moringa oleifera* leaves crude ethanolic extract and its fractions (butanol and ethyl acetate).

Peak no	t_R_ (min)	[M–H]^–^	[M + H]^+^	MS/MS	Annotation	Chemical class	Butanol fraction	Ethyl acetate fraction	Reference(s)
1	1.68	153	–	123, 109, 108, 91, 81, 80	Protocatechuic acid	Phenolic acids	–	+	^29,41^
2	1.81	353	–	191, 179, 161, 135	(neo)Chlorogenic acid		+ +	+	^35^
3	2.50	337	–	191, 163, 136, 119, 111	*p*–Coumaroylquinic acid		+ +	+	^29,35^
4	4.25	612	–	275, 148, 97, 75, 72	Acetyl–glucomoringin	Thioglycosides			
(Glucosinolates)	+	–	^31^						
5	4.88	313	–	161, 137, 121	Benzoylhydroxy ferulic acid	Phenolic acid	–	+	^36^
6	5.24	593	–	487, 353, 188	Vicenin–2	Flavonoid	+	–	^35^
7	5.45	324	–	235	Quinic acid pentoside*	Phenolic acid	–	+	^36^
8	6.35	463	–	300, 283, 151	Quercetin–*O*–hexoside	Flavonoids	+	+ +	^43^
9	6.70	447	–	284, 255, 227, 223, 211, 174, 151	Kaempferol–*O*–hexoside		+	+ +	^35^
10	6.77	187	–	187, 169, 125, 123, 97, 57	Gallic acid monohydrate	Phenolic derivative	–	+ + +	^43^
11	6.94	533	–	285, 284, 283, 211, 185, 165	Kaempferol–malonylglucoside	Flavonoids	+	+ +	^48^
12	7.00	–	332	332, 314, 286, 256, 191, 177, 148, 91, 86	Pentahydroxy–methoxy–flavone		+ +	+	^49^
13	8.82	301	–	255, 205, 179, 151, 149, 121, 107, 83	Ellagic acid or quercetin	Phenolic derivative	–	+	^50,51^
14	8.85	327	–	193, 183, 137, 97, 57, 43	Unidentified	–	–	+	–
15	9.44	329	–	272, 249	Dimethyl–O–ellagic acid	Phenolic derivative	–	+	^52^
16	9.79	431	–	145	Unidentified	–	+	–	–
17	9.85	–	181*	181, 163, 145, 139, 135, 121, 107, 93, 91, 69	Theobromine	Alkaloid	–	–	^53^
18	10.7	307*	–	185, 125, 121	Catechin hydrate	Flavonoid	–	–	^36^
19	11.3	431	–	311, 283, 222, 215, 113	Vitexin		+	–	^31,54,55^
20	12.2	609	–	300, 301	Rutin		+	–	^29,35^
21	13.6	721	–	622, 560, 415, 144, 125, 89	Di–O–caffeoyl–O–sinapoyl–quinic acid	Phenolic derivative	+	+	^56^
22	13.9	489	–	297, 284, 171	Kaempferol acetyl glycoside	Flavonoid	+	+	^29,31,57^
23	14.3	293*	–	275, 265, 235, 211, 183, 181, 131, 121, 81	Hydroxy octa–decatrienoic Acid	Hydroxy fatty acids	–	–	^31,36^
24	14.3	409	–	161, 135, 133, 127, 85	Unidentified	–	+	–	–
25	14.5	–	277*	185, 161, 149, 135, 121, 107, 93, 79, 69, 43	Hydroxy hepta–decapentaenoic acid	Hydroxy fatty acid	–	–	^36^
26	15.3	327	–	312	Caffeoyl tartaric acid hexoside	Phenolic derivative	–	+	^28^
27	15.5	295*	–	295, 195, 183, 113	Hydroxy octa–decadienoic acid	Hydroxy fatty acid	–	–	^36^
28	16.3	621	–	149, 89, 57	Unidentified	–	+	–	–
29	18.1	555	–	555, 255, 225, 206, 153	SQMG 16:0				
(sulfoquinovosyl monoacylglycerol)	Sulfolipids	+	–	^58,59^					
30	26.1	–	391	273, 227, 209, 183, 167, 149, 111	Diethylhexylphthalate (DEHP)	Phthalates	–	+	^30^

* Masses that were only detected in the ethanol extract, yet present in traces or neglected amounts in the butanol and ethyl acetate fractions.

Based on the relative abundance of mass ions in the fractions, they were given symbols +  +  + (highly abundant), +  + , + , and – (not present).

### Phenolic acids

Many phenolic acids were detected in the leaves of *Moringa oleifera*, including protocatechuic and chlorogenic acids, along with other quinic, ferulic, and caffeic acid derivatives. Protocatechuic acid (peak 1) displayed an [M-H]- peak at m/z 153 and a base peak fragment at m/z 109, which is formed due to decarboxylation (− 44 Da). (neo)Chlorogenic acid is a caffeoylquinic acid with a deprotonated ion peak at m/z 353, indicating a fragmentation transition typical of caffeic acid, such as m/z 179 [M- H-C7H10O5]-, which loses CO_2_ to form an ion at m/z 135. A base peak fragment at m/z 191 [M-H-C9H6O3]- is indicative of a deprotonated quinic acid moiety. Peak 3 was eluted at 2.5 min and showed [M-H]- ion at m/z 337 and a base peak fragment at m/z 163, indicative of 3-p-coumaroylquinoc acid based on the hierarchical scheme reported by Clifford et al.^26^**.** Compound 14 (tR = 9.44 min) displayed [M-H]-at m/z 329 and a fragment ion at m/z 272 [M-H-2CH3-CO]-, suggesting a dimethyl-O-ellagic acid. Peaks 20 and 25 were identified as dicaffeoyl sinapoyl quinic acid and caffeoyl tartaric acid hexoside, respectively, based on the similarity of their mass fragments to those previously reported in the literature^27,28^

### Flavonoids

Phenolic acids and flavonoids are the two main classes identified in the ethanolic extract of *Moringa oleifera*, the butanol fraction, and the ethyl acetate fraction. Among the identified flavonoids are vicenin-2 (Apigenin 6,8-di-C-glucoside), quercetin-O-hexoside, kaempferol-O-hexoside, kaempferol malonyl glucoside, pentahydroxy methoxyflavone, catechin hydrate, vitexin, rutin, and kaempferol acetyl glycoside. Two apigenin-C-glucosides were detected, including vicenin-2, which was identified based on bibliographic data^29^**,** and vitexin (peak 18, tR = 11.3 min) with characteristic fragments at m/z 311 [M-H-120]- and m/z 283 attributed to the aglycone part. Two quercetin derivatives were detected, including compound 8 (tR = 6.35 min) with [M-H]- ion at m/z 463, showing a neutral loss of hexose (-162Da ) and rutin (peak 19, tR = 12.2 min) with a base peak fragment attributed to quercetin aglycone. Three kaempferol derivatives were identified including peak 9 (tR = 6.70 min) with base peak fragment attributed to the loss of hexose, peak 10 (tR = 6.94 min) with base peak fragment formed after the loss of malonylglucoside (-249 Da), and peak 21 (tR = 13.9 min) with base peak fragment of kaempferol aglycone formed after the loss of acetyl glucoside.

### Hydroxy fatty acids

Three hydroxy fatty acids, eluting at the end of the chromatogram, were identified, including hydroxyoctadecatrienoic acid (tR = 14.3 min), hydroxyheptadecapentaenoic acid (tR = 14.5 min), and hydroxyoctadecadienoic acid (tR = 15.5 min). Their fragmentation is based on the successive loss of one or more water molecules and decarboxylation steps.

### GC–MS analysis of the hexane fraction of *Moringa oleifera*

More than 40 metabolites (representing nearly 45.79% of the total GC–MS chromatogram) were tentatively identified in the hexane fraction of *Moringa oleifera* based on matching their retention indices with those reported in the NIST database and the human metabolome database. They are classified into linear, cyclic, and aromatic hydrocarbons, as well as lactones, fatty acid esters, phthalate esters, and steroids (Table 7). Abundant components, based on their peak area, are shown in Figure S3 and include hexahydrofarnesyl acetone, palmitic acid ethyl ester, 2- 2-hexanecan-1-ol, 3,7,11,15-tetramethyl acetate, linolenic acid methyl ester, 4,8,12,16-tetramethylheptadecan-4-olide,1-dehydro-10-gingerdione, strophanthidol, 24-norursa-3,12-diene, and betulinaldehyde.

**Table 7:** GC–MS analysis of the hexane extract of *Moringa oleifera* leaves.

Peak no	t_R_ (min)	Area %	Annotation	Base *m/z*	KI_rep_	KI_exp_	Chemical class	SI (%)	Molecular formula
1	15.9	0.67	2,4-Dimethylundecane	85.15	1185	1194	Linear hydrocarbons	84	C_13_H_28_
2	16.8	0.18	1-Hexylcyclohexane	83.15	1224	1227	Cyclic hydrocarbons	80	C_12_H_24_
3	17.1	0.83	Methyltetralin	118.20	1246*	1235	Cyclic hydrocarbons	88	C_11_H_14_
4	18.1	0.51	5-Ethyl-5-methyldecane	113.20	1229	1269	Linear hydrocarbons	75	C_13_H_28_
5	18.7	0.25	(3-Methylcyclopentyl)benzene	104.15	1295	1289	Aromatic hydrocarbons	80	C_12_H_16_
6	18.8	0.16	2,4-Dimethyldodecane	85.15	1285	1293	Linear hydrocarbons	80	C_14_H_30_
7	19.8	0.12	*n*-Heptyl cyclohexane	83.15	1328	1330		79	C_13_H_26_
8	20.1	0.13	*n*-Heptylbenzene	92.15	1338	1339	Aromatic hydrocarbons	76	C_13_H_20_
9	22.3	0.35	*cis*-Geranylacetone	43.05	1420	1419	Oxygenated monoterpenes	81	C_13_H_22_O
10	23.6	0.53	Dihydroactinidiolide	111.15	1473	1471	Lactones	88	C_11_H_16_O_2_
11	24.0	0.11	2,4-di-*t*-Butylphenol	191.20	1512	1484	Phenols	91	C_14_H_22_O
12	24.2	0.13	7-(1,3-Dimethylbuta-1,3-dienyl)-1,6,6-trimethyl-3,8-dioxatricyclo[5.1.0.0(2,4)]octane	85.15	1473	1495	Cyclic ethers	75	C_15_H_22_O_2_
13	28.7	0.15	2-Phenylundecane	105.15	1692	1678	Aromatic hydrocarbons	94	C_17_H_28_
14	29.3	0.16	6-Phenyldodecane	91.10	1719	1706		79	C_18_H_30_
15	29.4	0.11	(1-Butylheptyl)benzene	91.10	1724	1710		84	C_17_H_28_
16	30.2	0.20	3-Phenylundecane	91.10	1724	1744		76	C_17_H_28_
17	30.7	0.21	Myristic acid, ethyl ester	88.10	1779	1768	Fatty acid esters	84	C_16_H_32_O_2_
18	31.2	0.18	3-Methyl-butyric acid, 4-butyl-2-methyl-5-oxotetrahydrofuran-3-yl ester	85.20	1807	1790	Lactones	79	C_14_H_24_O_4_
19	31.5	0.20	6-Phenyldodecane	91.10	1823	1804	Aromatic hydrocarbons	82	C_18_H_30_
20	31.7	0.18	5-Phenyldodecane	91.10	1823	1811		86	C_18_H_30_
21	31.9	1.82	Hexahydrofarnesyl acetone	43.05	1823	1820	Oxygenated sesquiterpenes	75	C_18_H_36_O
22	32.5	0.27	2-Methyl-7-octadecyne	82.15	1863	1849	Linear hydrocarbons	75	C_19_H_36_
23	32.9	0.33	2-Methyl-7-octadecyne	95.15	1863	1867		74	C_19_H_36_
24	33.2	0.17	2-Phenyltridecane	105.15	1894	1885	Aromatic hydrocarbons	92	C_19_H_32_
25	33.5	0.99	Palmitic acid, methyl ester	87.10	1878	1898	Fatty acid esters	83	C_17_H_34_O_2_
26	33.6	0.44	Di-sec-butyl phthalate	149.10	1908	1904	Phthalate esters	91	C_16_H_22_O_4_
27	34.7	0.49	9-Hexadecenoic acid, ethyl ester	96.15	1955	1956	Fatty acid esters	72	C_18_H_34_O_2_
28	34.9	2.04	Palmitic acid, ethyl ester	88.15	1978	1968		82	C_18_H_36_O_2_
29	36.5	0.14	Hexadecanoic acid, 4-hydroxy, γ-lactone	85.15	2104*	2049	Lactones	81	C_16_H_30_O_2_
30	36.8	0.47	Linolenic acid, methyl ester	79.10	2081	2063	Fatty acid esters	84	C_19_H_32_O_2_
31	36.9	0.29	(*Z*)-6-Octadecenoic acid, methyl ester	96.15	2085	2068		72	C_19_H_36_O_2_
32	37.3	2.28	2-Hexadecen-1-ol, 3,7,11,15-tetramethyl acetate	123.20	2168	2089		79	C_22_H_42_O_2_
33	37.5	0.20	Stearic acid, methyl ester	87.10	2077	2098		81	C_19_H_38_O_2_
34	38.0	0.95	1,16-Hexadecanediol	81.10	2130	2128	Fatty alcohol	73	C_16_H_34_O_2_
35	38.1	1.68	Linolenic acid, methyl ester	79.10	2101	2132	Fatty acid esters	82	C_19_H_32_O_2_
36	39.2	0.29	3,7,11,15-Tetramethylhexadec-2-en-1-yl acetate	123.20	2168	2192		77	C_22_H_42_O_2_
37	41.3	2.57	4,8,12,16-Tetramethylheptadecan-4-olide	99.15	2258	2311	Lactones	83	C_21_H_40_O_2_
38	41.6	0.32	n-Propyl linolenate	79.1	2300	2324	Fatty acid esters	81	C_21_H_36_O_2_
39	44.3	1.81	Tetracosane	85.2	2407	2488	Saturated alkanes	75	C_24_H_50_
40	44.4	3.12	Phthalic acid, di(6-methylhept-2-yl) ester	149.1	2475*	2494	Phthalate esters	90	C_24_H_38_O_4_
41	48.8	0.21	Octacosane	85.2	2804	2785	Saturated alkanes	71	C_28_H_58_
42	48.9	0.18	Squalene	81.1	2814*	2795		76	C_30_H_50_
43	49.3	11.21	1-Dehydro-10-gingerdione	43.1	2832*	2816	Phenolic	53	C_21_H_30_O_4_
44	50.4	0.66	1,1,6-trimethyl-3-methylene-2-(3,6,9,13-tetramethyl-6-ethenye-10,14-dimethylene-pentadec-4-enyl)cyclohexane	137.1	2885	2895	Hydrocarbons	72	C_33_H_56_
45	55.8	3.70	Strophanthidol	107.1	3238	3294	Steroids	73	C_23_H_34_O_6_
46	56.3	2.11	24-Norursa-3,12-diene	218.2	3105*	3328		84	C_29_H_46_
47	57.0	1.77	Betulinaldehyde	95.1	3628*	3377		78	C_30_H_48_O_2_
(%) Total identified compounds	45.79%								

* Reported on another non-polar column.

## Discussion

*Moringa oleifera* leaves have captivated the scientific community due to their potential for in vitro biological applications. properties. These include immune system modulation, thyroid hormone regulation, protection against gastric ulcers, anti-diabetic activity, hypotensive properties, anti-inflammatory, antibacterial, and anticancer activities, as well as the ability to improve liver and kidney function^4,5^. This study provides a comparative, solvent-dependent evaluation of *Moringa oleifera* leaf fractions derived from a single ethanolic extract, integrating biological screening with comprehensive chemical profiling. Rather than focusing on isolated bioactivities, the results demonstrate that each solvent fraction exhibits a distinct biological profile, including cytotoxic, antiviral, and antioxidant effects. Importantly, these fraction-specific activities correlate with the dominant phytochemical classes identified by LC–MS/MS and GC–MS analyses, highlighting the critical role of solvent polarity in shaping both chemical composition and biological performance. Collectively, these findings support the value of targeted fractionation as an exploratory strategy to elucidate the multifunctional bioactivity of complex plant extracts under in vitro conditions.

The cytotoxicity of the tested samples was evaluated at concentrations ranging from 0 to 1000 µg/mL against a panel of cancer cell lines: CA-549, MCF-7, HepG-2, Panc-1, Caco-2, and HELA. The superior potential in vitro biological action of the hexane fraction exhibited the highest cytotoxic potency, particularly against MCF-7 and HepG-2, with comparatively low toxicity against PBMCs (IC₅₀ = 93.27 µg/mL), this high potency can be attributed to the lipophilic nature of the hexane fraction, which, as revealed by GC–MS analysis, is enriched with fatty acids and sterols (e.g., β-sitosterol). Quantitatively, our hexane fraction demonstrated a potent IC₅₀ = 14.81 µg/mL against MCF-7 cells. This result shows superior efficacy compared to the study by Al-Asmari et al. (2015), who reported that the hexane extract of Moringa oleifera leaves exhibited an IC₅₀ = 230 µg/mL against the same MCF-7 cell line. This significant 11-fold increase in potency in our findings can be attributed to the enhanced recovery of specific lipophilic terpenoids and the geographic variation in the Egyptian chemotype, which appears to yield a more concentrated profile of cytotoxic metabolites compared to the samples from different agro-ecological zones reported in earlier studies^30^.

These non-polar compounds possess high membrane permeability, allowing them to effectively penetrate the lipid bilayer of cancer cells and interact with intracellular targets^31,32^. This selectivity mirrors earlier reports of *Moringa’s* anticancer effects. For instance, Pop et al.^9^ documented apoptosis induction in cancer cells via modulation of *BAX/BCL-2,* consistent with our qRT-PCR data showing *BAX* upregulation (e.g., 19.1-fold in breast carcinoma) and *BCL-2* downregulation (0.06-fold) after hexane fraction treatment. Notably, while the modulation of BAX and BCL-2 provides strong evidence of apoptosis at the mRNA level, the absence of protein-level validation (e.g., Western blot) is a study limitation that should be addressed in future work. Likewise, Krishnamurthy et al. reported significant cytotoxicity of the hexane fraction of *Moringa oleifera* against breast and liver cancer cell lines, indicating that lipophilic metabolites play a central role in cytotoxicity^10^. The induction of apoptosis, evidenced in our study by the upregulation of pro-apoptotic *BAX* and downregulation of anti-apoptotic *BCL-2*, concurs with findings from Golestani Eimani et al.^33^**,** who reported similar apoptotic gene modulation following treatment with flavonoid-rich plant extracts. Notably, the efficacy of the hexane fraction against HepG-2 and MCF-7 cells aligns with Talib et al*.,* who attributed *Moringa’s* anticancer activity to glucosinolates and flavonoids^6^. However, our GC–MS analysis identified hexahydrofarnesyl acetone (11.21%) and phytol derivatives (e.g., 2-hexadecen-1-o1,3,7,11,15–tetramethylhexadecyl acetate) as major constituents, suggesting that terpenoids and fatty acid esters contribute to its potency—a divergence from seed-focused studies that emphasize glucosinolates. Geographic differences may explain this: Nigerian *Moringa oleifera* hexane fractions prioritized phthalates^34^, while Egyptian samples included lactones (4,8,12,16-tetramethylheptadecan-4-olide) and steroids (strophanthidol). This highlights the impact of agro-ecological conditions on metabolite profiles and bioactivity.

In comparison, the ethanolic *Moringa oleifera* extract showed moderate, balanced activity across all cell lines. In contrast, the butanol fraction exhibited the weakest cytotoxic effect, which is consistent with its relatively low levels of flavonoids and fatty acids, as determined by LC–MS/MS analysis. This indicates that phenolic acids and flavonoids, which are more abundant in the hexane and ethyl acetate fractions, are largely responsible for cytotoxicity^35–37^.

A crucial aspect of any potential lead compound is its safety profile. In this study, we evaluated the Selectivity Index (SI) to benchmark the toxicity of the fractions against normal human PBMCs. The hexane fraction displayed a high SI (> 2) for both MCF-7 and HepG2 lines, suggesting a favorable window of selectivity. This broad-spectrum selectivity suggests that the lipophilic compounds in the hexane fraction target common oncogenic mechanisms prevalent in diverse solid tumors. Furthermore, the ethanolic extract demonstrated remarkably high SI values (ranging from 10.1 to 21.0), further reinforcing the therapeutic potential of the whole leaf matrix.

The antiviral activities of the ethanolic *Moringa oleifera* extract and its solvent fractions were tested in vitro against Hepatitis A virus HAV, HSV-1, and HSV-2. The butanol fraction demonstrated the most potent antiviral activity against HAV (IC₅₀ = 24.42 µg/mL, SI = 7.21), HSV-1/2 (IC₅₀ = 13.23–15.15 µg/mL, SI = 11.63–13.32), and all three viruses with a high SI value. To assess the safety of utilizing a compound as an antiviral agent, the selectivity index (SI) is a commonly used parameter. SI determines the difference between the cytotoxic and antiviral activities. Higher SI values indicate that drugs are more effective and safer to use^46^. To eliminate the virus at a concentration that would not damage host cells, the ideal medication would destroy the virus at low concentrations and the cells at high concentrations^38,39^. These values surpass the tested samples and approach the efficacy of the positive control, acyclovir (SI for HSV-1 = 45.12). These findings are consistent with those of Giugliano et al., who observed that *Moringa oleifera* leaf extract significantly inhibited the replication of respiratory and HSV-1 viruses and modulatedhe host immune response^8^. Regarding antiviral activity, our butanol fraction IC₅₀ = 13.23 µg/mL against HSV-1 showed significantly higher potency compared to the findings of Lipipun et al.^40^, who reported that polar extracts of Moringa oleifera exhibited an IC₅₀ 100 µg/mL against HSV-1. This difference arises from the solvent-specific enrichment of polar flavonoids (e.g., vicenin-2) in our butanol fraction, which are less concentrated in the crude polar extracts used in previous studie. While the SI values for *Moringa oleifera* fractions are significant, they remain preliminary. Unlike the reference drug Acyclovir, which showed an SI for HSV-1 = 45.12, our fractions represent a complex mixture of metabolites. Therefore, these results should be interpreted as a primary in vitro screening. Future studies are required to determine whether the observed effects are virucidal (extracellular) or involve inhibition of intracellular replication.

LC–MS/MS revealed that the butanol fraction was enriched in flavonoids (e.g., vicenin-2, kaempferol acetyl glycoside) and phenolic acids (chlorogenic acid), which are known to inhibit viral enzymes and modify cellular redox states^31,32^. As suggested by Dogan et al. in similar models, these polyphenols may enhance host cell antiviral defences by modulating the IFN-β response or interfering with viral glycoproteins^17^. Significant antiviral activity was also demonstrated by the ethyl acetate fraction, specifically against HSV-1 (IC₅₀ = 14.75 µg/mL, selectivity index [SI] = 13.63). This confirms previous studies that found quercetin derivatives as inhibitors of herpes simplex virus polymerase and helicase-primase complexes^41^. The ethanolic *Moringa oleifera* extract and methylene chloride fraction showed weak to no antiviral activity, indicating that bioactivity is not only phenol-dependent but also fraction-specific. In contrast, Vergara-Jimenez et al*.* highlighted the importance of targeted fractionation, broadly attributing *Moringa’s* antiviral properties to its phenolic antioxidants^5^

Compounds known as antioxidants protect cells from the damage caused by unstable molecules such as free radicals. The ethyl acetate fraction showed the highest antioxidant activity (IC₅₀ = 14.66 µg/mL) based on DPPH radical scavenging. This is much higher than the reference antioxidant ascorbic acid (IC₅₀ = 10.21 µg/mL). This aligns with a previous study that showed the phenolic and flavonoid-rich fractions of *Moringa oleifera* leaf extract to have high antioxidant potential^42^. These antioxidants play a critical role in decreasing oxidative stress, a known contributor to viral replication and cancer cell proliferation^5^. The methylene chloride fraction’s low activity (IC₅₀ = 459.39 µg/mL) suggests that non-polar solvents extract fewer antioxidants, which aligns with previous findings^41^. Moreover, the LC–MS/MS analysis performed in the current study identified flavonoids, including quercetin-O-hexoside and kaempferol glucosides, in the ethyl acetate fraction. These compounds are known for their potent ROS-scavenging activities and synergy with other polyphenols^43,44^. On the other hand, since the hexane fraction lacks these hydrogen-donating groups, its antioxidant capacity remains limited.

Apoptotic Gene Modulation: Ethanolic *Moringa oleifera* extract, ethyl acetate, and hexane fractions extensively modulated *BAX/BCL-2* expression in liver, breast, and pancreatic carcinoma cells. The hexane fraction induced the most substantial pro-apoptotic change, 12.29-fold *BAX* upregulation in liver carcinoma, while *Bcl* resulted in only 0.223-fold downregulation, likely due to its terpenoid and steroid components (e.g., betulinaldehyde, 24-norursa-3,12-diene). These results are supported by Golestani Eimani et al*.*^33^, who reported that plant-derived flavonoids and fatty acids increase mitochondrial apoptosis by modulating BAX/BCL-2.

Interestingly, the ethyl acetate fraction exhibited a strong pro-apoptotic gene expression profile, particularly in breast cancer cells, despite also showing moderate cytotoxicity. It contains abundant flavonoids, such as quercetin glycosides and kaempferol, which are known to induce apoptosis via ROS-mediated signalling pathways^9^.

The LC–MS/MS and GC–MS profiles, which revealed over 60 metabolites across fractions, show good correlation with the observed bioactivities. Phenolic acids and flavonoids, which have been shown to exhibit multifunctional bioactivity, including the induction of apoptosis, antioxidant properties, and antiviral effects, were abundant in the ethyl acetate fraction^9,45^. In contrast, the hexane fraction contained non-polar compounds, such as phytosterols, terpenoids, and hydroxy fatty acids, which contributed to its cytotoxicity but weak antiviral and antioxidant properties. These findings highlight that fraction polarity directly affects the potential in vitro biological activity profile of plant extracts, and that targeted extraction can optimise bioactivity for specific medical applications. Previous GC–MS of the hexane extract of *Moringa oleifera* from Palestine showed an abundance of fatty acids, especially stearic and palmitic acids^37^. Another study from India revealed the richness of the dichloromethane extract of *Moringa oleifera* in palmitic acid and cis-vaccenic acid^24^. In contrast, Jamiu et al*.*reported on the abundance of 2-heptanol, 6-methyl-1,2-benzenedicarboxylic acid, phthalic acid, cyclobutyl-heptyl ester, diethylphthalate, ethylisopropyl ester, trimethyl(4-tert.-butyl-phenoxy)silane, and 2,4,6-cycloheptatrien-1-one in the hexane and ethyl acetate fractions from Nigeria^34^. Differences in metabolites of the same plant species could be attributed to geographical distribution and agricultural conditions.

In summary, while the present findings highlight the in vitro potential of *moringa oleifera* fractions, particularly the hexane fraction’s pro-apoptotic activity, these results should be interpreted as exploratory. The transition from in vitro observations to clinical application involves significant physiological complexities. Therefore, further studies, including in vivo models and pharmacokinetic evaluations, are required to evaluate their efficacy and safety in complex biological systems.

## Conclusion

Based on solvent-specific phytochemical clusters, this study confirms the cytotoxic, antioxidant, and antiviral characteristics of *Moringa oleifera* ethanolic extract and its fractions. Potent selective cytotoxicity was highest in the hexane fraction. The strongest activity, particularly against HepG-2 and MCF-7, while showing minimal toxicity to normal PBMCs, underscores its potential in vitro biological functions. safety and suggests tumor-selective mechanisms probably mediated by terpenoids and fatty acid esters. This effect was further supported by the upregulation of pro-apoptotic *BAX* and downregulation of anti-apoptotic *BCL-2,* indicating induction of programmed cell death. The butanol fraction displayed remarkable antiviral effects against HAV and HSV-1/2, with a high selectivity index, whereas the ethyl acetate fraction showed the strongest antioxidant capacity, likely due to its high content of phenolic acids. Our findings, supported by the identification of over 60 bioactive compounds using LC–MS/MS and GC–MS, provide a scientific basis for further development of these fractions either into targeted phytopharmaceuticals or broader therapeutic applications were a standardized enriched extract that combines several fractions can provide a multitarget therapy. 

## Supplementary Information

Below is the link to the electronic supplementary material.

Supplementary Material 1

## Data Availability

All data generated or analyzed during this study are included in this published article and supplementary file.

## References

[1] Verma, S. & Singh, S. Current and future status of herbal medicines. *Vet. World* **2**, 347 (2008).

[2] Atanasov, A. G. et al. Natural products in drug discovery: Advances and opportunities. *Nat. Rev. Drug Discov.* **20**, 200–216. 10.1038/s41573-020-00114-z (2021). 33510482 10.1038/s41573-020-00114-z PMC7841765

[3] Pareek, A. et al. *Moringa oleifera*: An updated comprehensive review of its pharmacological activities, ethnomedicinal, phytopharmaceutical formulation, clinical, phytochemical, and toxicological aspects. *Int. J. Mol. Sci.* **24**(3), 2098. 10.3390/ijms24032098 (2023). 36768420 10.3390/ijms24032098 PMC9916933

[4] Klimek-Szczykutowicz, M. *et al. Moringa oleifera* (drumstick tree)—nutraceutical, cosmetological and medicinal importance: a review. *Front Pharmacol* **15**, (2024). 10.3389/fphar.2024.1288382 PMC10869624 38370483

[5] Vergara-Jimenez, M., Almatrafi, M. & Fernandez, M. Bioactive components in *Moringa oleifera* leaves protect against chronic disease. *Antioxidants* **6**, 91 (2017). 29144438 10.3390/antiox6040091 PMC5745501

[6] Talib, W. H. et al. Immunomodulatory and anticancer effects of moringa polyherbal infusions: Potentials for preventive and therapeutic use. *Front. Immunol.*10.3389/fimmu.2025.1597602 (2025). 40612957 10.3389/fimmu.2025.1597602 PMC12221896

[7] Chhikara, N. et al. Exploring the nutritional and phytochemical potential of sorghum in food processing for food security. *Nutr. Food Sci.* **49**, 318–332 (2019).

[8] Giugliano, R. et al. Antiviral properties of *Moringa oleifera* leaf extracts against respiratory viruses. *Viruses* **16**, 1199 (2024). 39205173 10.3390/v16081199 PMC11359668

[9] Pop, O. L., Kerezsi, A. D. & Ciont, C. N. A comprehensive review of *Moringa oleifera* bioactive compounds—Cytotoxicity evaluation and their encapsulation. *Foods* **11**, 3787 (2022). 36496595 10.3390/foods11233787 PMC9737119

[10] Krishnamurthy, P. T., Vardarajalu, A., Wadhwani, A. & Patel, V. Identification and characterization of a potent anticancer fraction from the leaf extracts of *Moringa oleifera* L.. *Indian J. Exp. Biol.* **53**, 98–103 (2015). 25757240

[11] Moyo, B., Oyedemi, S., Masika, P. J. & Muchenje, V. Polyphenolic content and antioxidant properties of *Moringa oleifera* leaf extracts and enzymatic activity of liver from goats supplemented with *Moringa oleifera* leaves/sunflower seed cake. *Meat Sci.* **91**, 441–447 (2012). 22465510 10.1016/j.meatsci.2012.02.029

[12] Elhusseiny, S. M. et al. Antiviral, cytotoxic, and antioxidant activities of three edible agaricomycetes mushrooms: *Pleurotus columbinus*, *Pleurotus sajor-caju*, and *Agaricus bisporus*. *J. Fungi*10.3390/jof7080645 (2021). 10.3390/jof7080645 PMC8399653 34436184

[13] El Sayed, O. H. et al. Production of hydroxy marilone C as a bioactive compound from *Streptomyces badius*. *J. Genet. Eng. Biotechnol.* **14**, 161–168 (2016). 30647610 10.1016/j.jgeb.2016.04.001 PMC6299901

[14] Feoktistova, M., Geserick, P. & Leverkus, M. Crystal Violet Assay for Determining Viability of Cultured Cells. *Cold Spring Harb Protoc* **2016**, pdb.prot087379 (2016). 10.1101/pdb.prot087379 27037069

[15] Vijayan, P., Raghu, C., Ashok, G., Dhanaraj, S. A. & Suresh, B. Antiviral activity of medicinal plants of Nilgiris. *Indian J. Med. Res.* **120**, 24–29 (2004). 15299228

[16] Freimoser, F. M., Jakob, C. A., Aebi, M. & Tuor, U. The MTT [3-(4,5-Dimethylthiazol-2-yl)-2,5-Diphenyltetrazolium Bromide] assay is a fast and reliable method for colorimetric determination of fungal cell densities. *Appl. Environ. Microbiol.* **65**, 3727–3729 (1999). 10427074 10.1128/aem.65.8.3727-3729.1999 PMC91559

[17] Doğan, H. H., Karagöz, S. & Duman, R. In vitro evaluation of the antiviral activity of some mushrooms from Turkey. *Int. J. Med. Mushrooms.* **20**, 201–212 (2018). 29717666 10.1615/IntJMedMushrooms.2018025468

[18] Lu, C., Li, H., Li, C., Chen, B. & Shen, Y. Chemical composition and radical scavenging activity of *Amygdalus pedunculat*a Pall leaves’ essential oil. *Food Chem. Toxicol.* **119**, 368–374 (2018). 29432838 10.1016/j.fct.2018.02.012

[19] Elhusseiny, S. M. et al. Proteome analysis and in vitro antiviral, anticancer and antioxidant capacities of the aqueous extracts of *Lentinula edodes* and *Pleurotus ostreatus* edible mushrooms. *Molecules*10.3390/molecules26154623 (2021). 34361776 10.3390/molecules26154623 PMC8348442

[20] Cao, H. & Shockey, J. M. Comparison of TaqMan and SYBR Green qPCR methods for quantitative gene expression in Tung Tree tissues. *J. Agric. Food Chem.* **60**, 12296–12303 (2012). 23176309 10.1021/jf304690e

[21] Eltokhy, M. A. et al. Exploring the nature of the antimicrobial metabolites produced by *Paenibacillus ehimensis* soil isolate mz921932 using a metagenomic nanopore sequencing coupled with lc-mass analysis. Antibiotics 11, (2022). 10.3390/antibiotics11010012 PMC8773065 35052889

[22] Eltokhy, M. A. et al. A metagenomic nanopore sequence analysis combined with conventional screening and spectroscopic methods for deciphering the antimicrobial metabolites produced by *Alcaligenes faecali*s soil isolate MZ921504. *Antibiotics*10.3390/antibiotics10111382 (2021). 34827320 10.3390/antibiotics10111382 PMC8614704

[23] Rahayu, I. & Timotius, K. H. Phytochemical analysis, antimutagenic and antiviral activity of *Moringa oleifera* L. leaf infusion: In vitro and in silico studies. *Molecules* **27**, 4017 (2022). 35807260 10.3390/molecules27134017 PMC9268431

[24] Navarro, R. R., Ichikawa, H., Iimura, Y. & Tatsumi, K. Removal of polycyclic aromatic hydrocarbons from contaminated soil by aqueous DNA solution. *Environ. Sci. Technol.* **41**, 4240–4245 (2007). 17626419 10.1021/es0624523

[25] Khandelwal, S. & Khurana, S. M. P. Molecular docking studies and GC-MS analysis of the antimicrobial compounds isolated from leaves of *Moringa oleifer*a. *Med. Plants.* **11**, 95 (2019).

[26] Clifford, M. N., Johnston, K. L., Knight, S. & Kuhnert, N. Hierarchical scheme for LC-MS n identification of chlorogenic acids. *J. Agric. Food Chem.* **51**, 2900–2911 (2003). 12720369 10.1021/jf026187q

[27] Ehuwa, O., Jaiswal, A. K. & Jaiswal, S. Salmonella, food safety and food handling practices. *Foods* **10**, 907 (2021). 33919142 10.3390/foods10050907 PMC8143179

[28] Kargbo, R., Onivogui, G. & Song, Y. In vitro anti-diabetic activity and phenolic compound profile of ethanol extracts of *Anisophyllea laurina* R. Br. ex Sabine leaves and stem bark. *Eur. Acad. Res.* **2**, 16089–16106 (2015).

[29] Wang, J. et al. LC-MS/MS-based chemical profiling of water extracts of *Moringa oleifera* leaves and pharmacokinetics of their major constituents in rat plasma. *Food Chem. X* **23**, 101585. 10.1016/j.fochx.2024.101585 (2024). 39027684 10.1016/j.fochx.2024.101585 PMC11255104

[30] Al-Asmari, A. K. et al. *Moringa oleifera* as an anti-cancer agent against breast and colorectal cancer cell lines. *PLoS ONE* **10**(8), e0135814. 10.1371/journal.pone.0135814 (2015). 26288313 10.1371/journal.pone.0135814 PMC4545797

[31] Yan, G., Sun, L. & Yongliang, Z. UPLC-Q-Orbitrap-MS2 analysis of *Moringa oleifera* leaf extract and its antioxidant, antibacterial and anti-inflammatory activities. *Nat. Prod. Res.* **34**(14), 2090–2094. 10.1080/14786419.2019.1573237 (2020). 30810361 10.1080/14786419.2019.1573237

[32] Zakaryan, H., Arabyan, E., Oo, A. & Zandi, K. Flavonoids: Promising natural compounds against viral infections. *Arch. Virol.* **162**, 2539–2551 (2017). 28547385 10.1007/s00705-017-3417-y PMC7087220

[33] Golestani Eimani, B. et al. Expression and prognostic significance of bcl-2 and bax in the progression and clinical outcome of transitional bladder cell carcinoma. *Cell J.* **15**, 356–363 (2014). 24381861 PMC3866540

[34] Jamiu, A. M. et al. Nutritional Values and Effects of Selected Solvents on the Phytochemical Properties of *Moringa Oleifera* Leaves Obtained from Sokoto, Nigeria. in 2024 International Conference on Science, Engineering and Business for Driving Sustainable Development Goals (SEB4SDG) 1–7 (IEEE, 2024). 10.1109/SEB4SDG60871.2024.10630192.

[35] Llorent-Martínez, E. J. et al. Preliminary phytochemical screening and antioxidant activity of commercial *Moringa oleifera* food supplements. *Antioxidants*10.3390/antiox12010110 (2023). 36670972 10.3390/antiox12010110 PMC9855063

[36] Abdel Shakour, Z. T. et al. Dissection of *Moringa oleifera* leaf metabolome in context of its different extracts, origin and in relationship to its biological effects as analysed using molecular networking and chemometrics. *Food Chem.* **399**, 133948. 10.1016/j.foodchem.2022.133948 (2023). 35994855 10.1016/j.foodchem.2022.133948

[37] Anzano, A. et al. Chemical analysis and antimicrobial activity of *Moringa oleifera* Lam. leaves and seeds. *Molecules* **27**, 8920 (2022). 36558052 10.3390/molecules27248920 PMC9782826

[38] Anderson, O. et al. An investigation of the antileishmanial properties of semi-synthetic saponins. *RSC Med. Chem.* **11**, 833–842 (2020). 33479679 10.1039/d0md00123f PMC7651632

[39] Zubair, M. S., Khairunisa, S. Q., Widodo, A. & Pitopang, R. Antiviral screening on *Alpinia eremochlamys, Etlingera flexuosa,* and *Etlingera acanthoides* extracts against HIV-infected MT-4 cells. *Heliyon* **7**, e06710 (2021). 33869876 10.1016/j.heliyon.2021.e06710 PMC8045043

[40] Lipipun, V. et al. Efficacy of Thai medicinal plant extracts against herpes simplex virus type 1 infection in vitro and in vivo. *Antivir. Res.* **60**(3), 175–180. 10.1016/s0166-3542(03)00152-9 (2003). 14638393 10.1016/s0166-3542(03)00152-9

[41] Mutungi, M. M. et al. Antioxidant and antiproliferative potentials of *Ficus glumosa* and its bioactive polyphenol metabolites. *Pharmaceuticals*10.3390/ph14030266 (2021). 33804242 10.3390/ph14030266 PMC8001017

[42] El-Sherbiny, G. M., Alluqmani, A. J., Elsehemy, I. A. & Kalaba, M. H. Antibacterial, antioxidant, cytotoxicity, and phytochemical screening of *Moringa oleifera* leaves. *Sci. Rep.* **14**, 30485 (2024). 39681592 10.1038/s41598-024-80700-y PMC11649684

[43] Kang, J. et al. Identification and characterization of phenolic compounds in hydromethanolic extracts of sorghum wholegrains by LC-ESI-MS(n). *Food Chem.* **211**, 215–226. 10.1016/j.foodchem.2016.05.052 (2016). 27283625 10.1016/j.foodchem.2016.05.052

[44] Granafei, S. et al. Unambiguous regiochemical assignment of sulfoquinovosyl mono- and diacylglycerols in parsley and spinach leaves by liquid chromatography/electrospray ionization sequential mass spectrometry assisted by regioselective enzymatic hydrolysis. *Rapid Commun. Mass Spectrom.* **31**(18), 1499–1509. 10.1002/rcm.7928 (2017). 28657161 10.1002/rcm.7928

[45] Ghali, R. et al. LC‑MS profiling and antioxidant, antifungal, and anticancer potentials of Tunisian *Allium sativu*m L. extracts. *PLoS ONE* **20**(6), e0325227. 10.1371/journal.pone.0325227 (2025). 40489471 10.1371/journal.pone.0325227 PMC12148127

[46] Randazzo, W., Piqueras, J., Rodríguez-Díaz, J., Aznar, R. & Sánchez, G. Improving efficiency of viability-qPCR for selective detection of infectious HAV in food and water samples. *J. Appl. Microbiol.* **124**, 958–964 (2018). 28649706 10.1111/jam.13519

[47] Li, P. et al. Upregulated miR-106a plays an oncogenic role in pancreatic cancer. *FEBS Lett.* **588**, 705–712 (2014). 24444603 10.1016/j.febslet.2014.01.007

[48] Oldoni, T. L. C. et al. Bioguided extraction of phenolic compounds and UHPLC-ESI-Q-TOF-MS/MS characterization of extracts of *Moringa oleifera* leaves collected in Brazil. *Food Res. Int.* **125**, 108647. 10.1016/j.foodres.2019.108647 (2019). 31554035 10.1016/j.foodres.2019.108647

[49] Ponce, Ma. A. et al. Flavonoids from shoots and roots of *Trifolium repens* (white clover) grown in presence or absence of the arbuscular mycorrhizal fungus *Glomus intraradices*. *Phytochemistry* **65**(13), 1925–1930. 10.1016/j.phytochem.2004.06.005 (2004). 15279999 10.1016/j.phytochem.2004.06.005

[50] Zhu, Y., Yin, Q. & Yang, Y. Comprehensive investigation of *Moringa oleifera* from different regions by simultaneous determination of 11 polyphenols using UPLC-ESI-MS/MS. *Molecules*10.3390/molecules25030676 (2020). 32033309 10.3390/molecules25030676 PMC7037984

[51] Grati, W. et al. HESI-MS/MS analysis of phenolic compounds from *Calendula aegyptiaca* fruits extracts and evaluation of their antioxidant activities. *Molecules*10.3390/molecules27072314 (2022). 35408713 10.3390/molecules27072314 PMC9000822

[52] Abdel Ghani, A. E. et al. UPLC-ESI-MS/MS profiling and cytotoxic, antioxidant, anti-inflammatory, antidiabetic, and antiobesity activities of the non-polar fractions of *Salvia hispanica* L. aerial parts. *Plants*10.3390/plants12051062 (2023). 36903922 10.3390/plants12051062 PMC10005563

[53] Hamed, M. M. et al. UPLC-ESI-MS analysis of secondary metabolites from *Tamarix aphylla* with its in-vitro anti-inflammatory and anti-microbial activities. *Egypt. J. Chem.* **67**(9), 1–12. 10.21608/EJCHEM.2024.271590.9403 (2024).

[54] Ashraf, H. et al. UPLC-ESI/MS/MS profiling and anti-inflammatory activity of *Gleditsia caspica*. *Arch. Pharm. Sci. Ain Shams Univ.* **4**(1), 124–134. 10.21608/APS.2020.2004.1042 (2020).

[55] Mohamed Yunus, S. N. et al. Antioxidant and α-glucosidase inhibitory activities of eight neglected fruit extracts and UHPLC-MS/MS profile of the active extracts. *Food Sci. Biotechnol.* **30**(2), 195–208. 10.1007/s10068-020-00856-x (2021). 33732510 10.1007/s10068-020-00856-x PMC7914318

[56] Jaiswal, R. & Kuhnert, N. Hierarchical scheme for liquid chromatography/multi-stage spectrometric identification of 3,4,5-triacyl chlorogenic acids in green *Robusta coffee* beans. *Rapid Commun. Mass Spectrom.* **24**(15), 2283–2294. 10.1016/j.sajb.2021.07.008 (2010). 20607843 10.1002/rcm.4639

[57] Teclegeorgish, Z. W. et al. Nutrients, secondary metabolites and anti-oxidant activity of *Moringa oleifera* leaves and Moringa-based commercial products. *S. Afr. J. Bot.* **142**, 409–420 (2021).

[58] Keusgen, M. et al. Sulfoquinovosyl diacylglycerols from the alga *Heterosigma carterae*. *Lipids* **32**(10), 1101–1112. 10.1007/s11745-997-0142-9 (1997). 9358437 10.1007/s11745-997-0142-9

[59] Rakkestad, K. et al. Phthalate levels in Norwegian indoor air related to particle size fraction. *J. Environ. Monit.* **9**, 1419–1425. 10.1039/b709947a (2008). 10.1039/b709947a 18049782

