# Supplementary material for: Bioactivity-guided analysis of Moringa olifera fractionated extracts for potential medical application
Source: Sci Rep. 2026 Mar 24;16:9939. doi: 10.1038/s41598-026-42314-4 (PMC13022387; doi:10.1038/s41598-026-42314-4)
Supplement: Supplementary file 1 — Supplementary Material 1 [file 41598_2026_42314_MOESM1_ESM.docx]

| **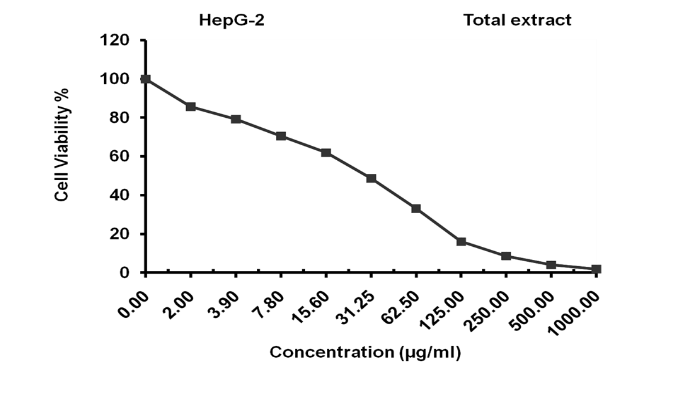** | **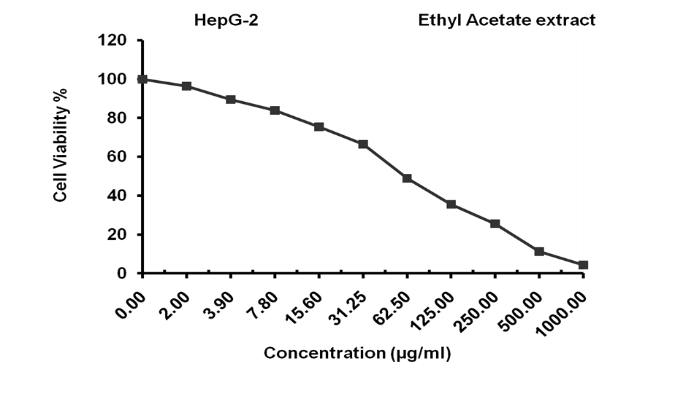** |
| --- | --- |
| **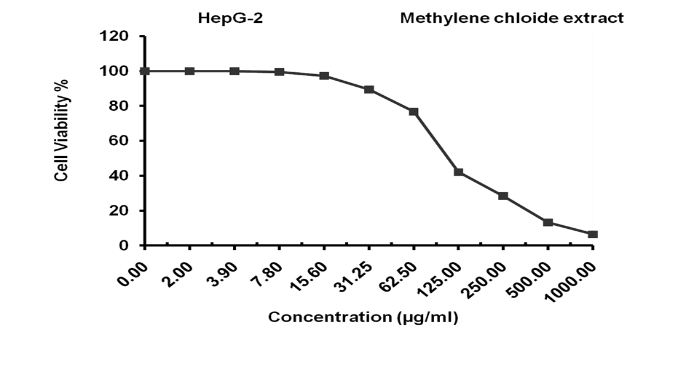** | **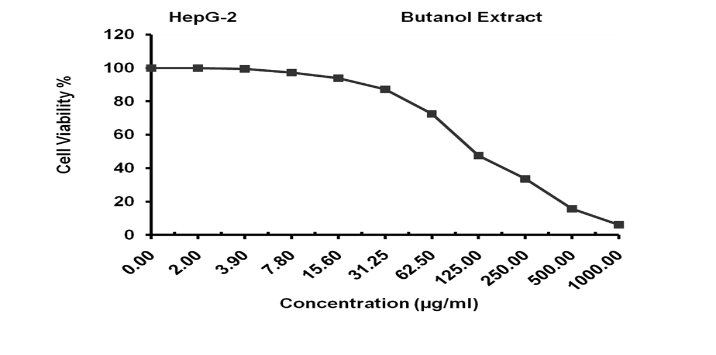** |
| **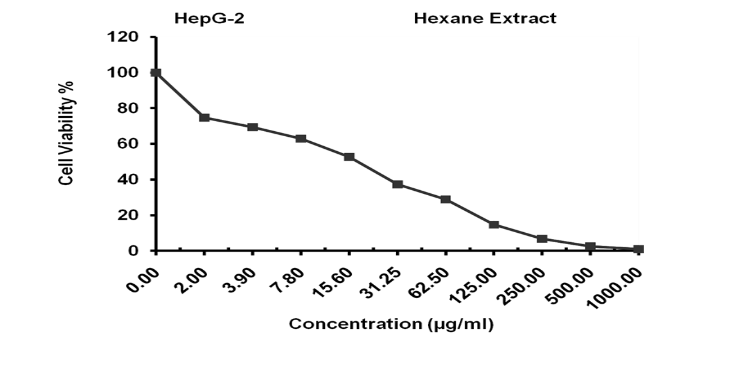** | **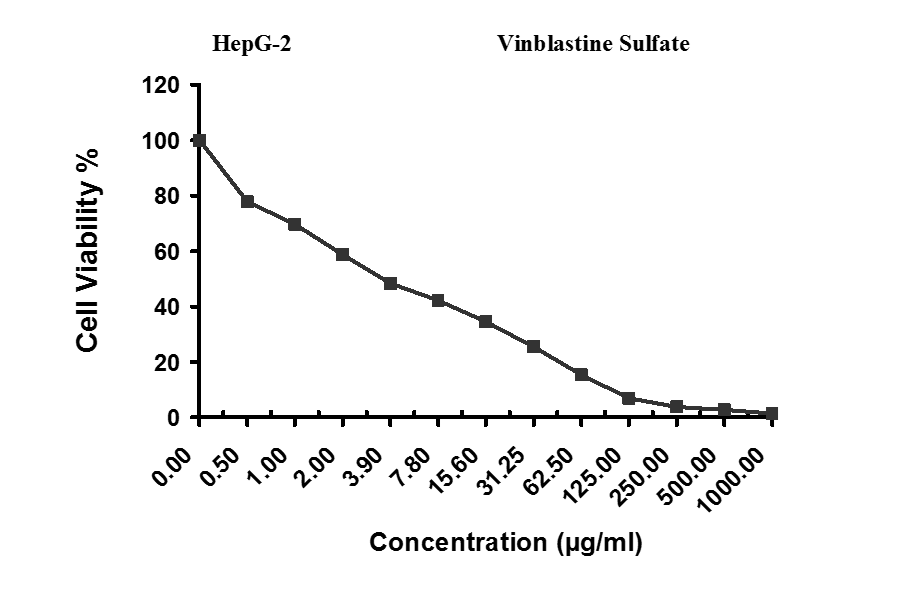** |
| **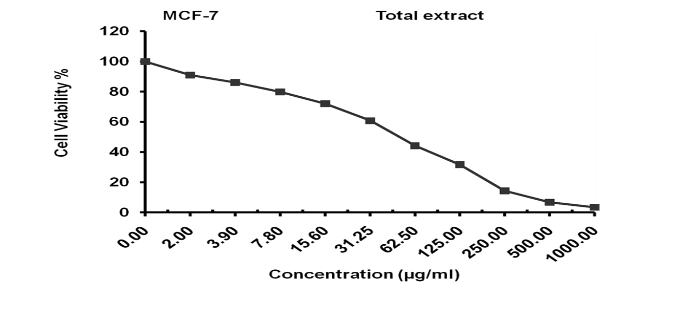** | **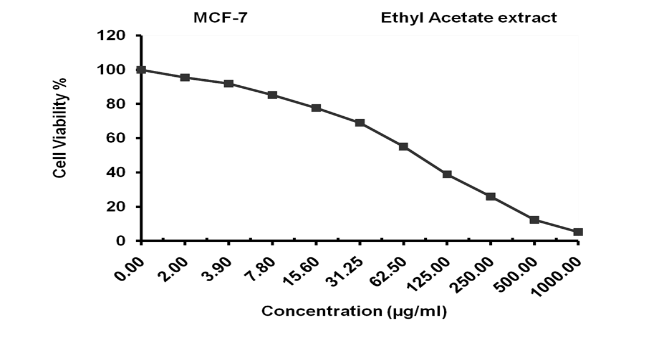** |
| **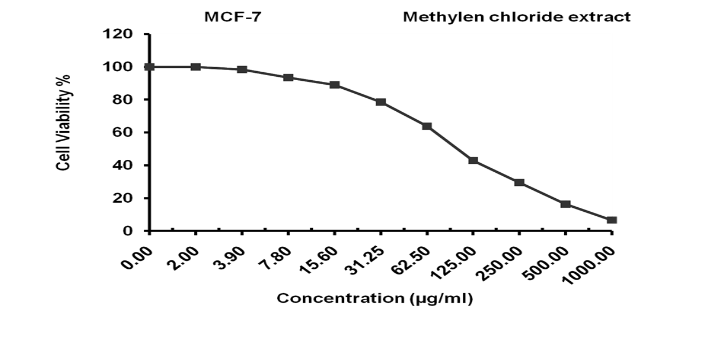** | **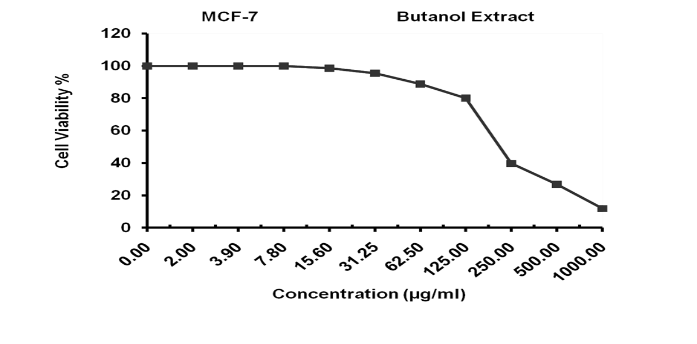** |
| **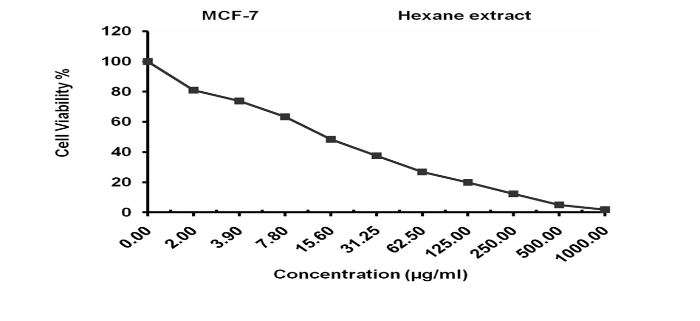** | **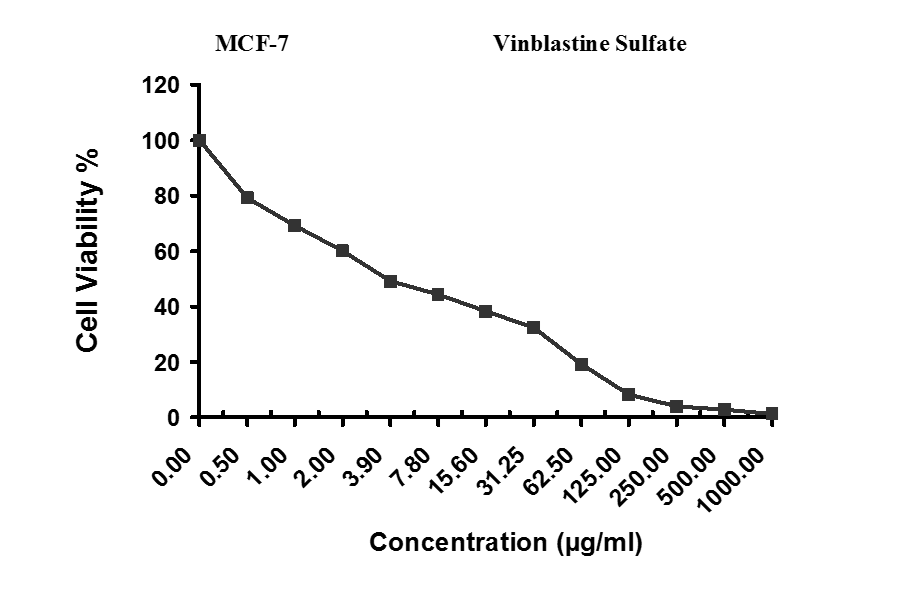** |
| **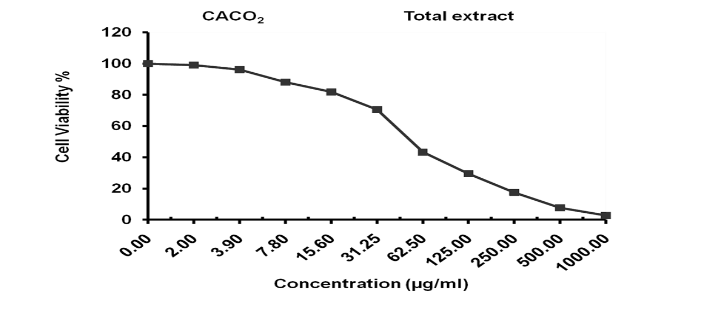** | **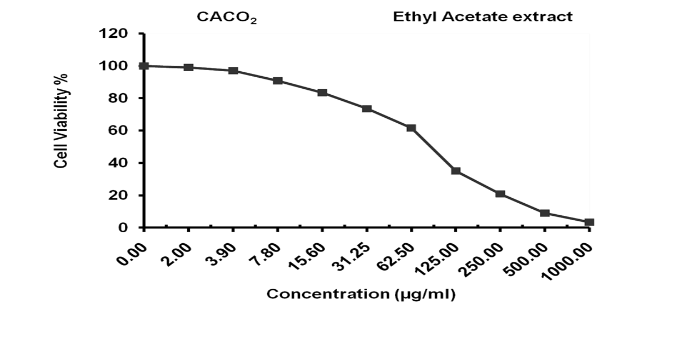** |
| **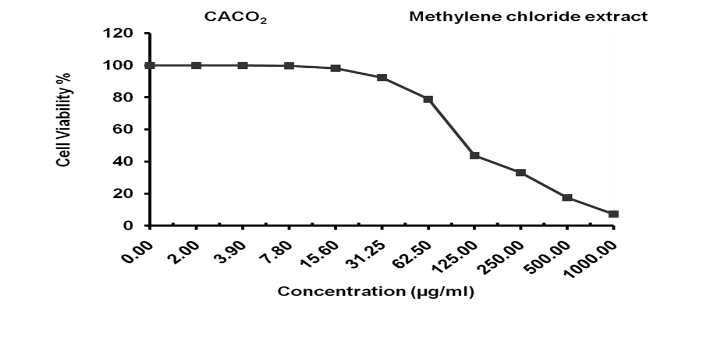** | **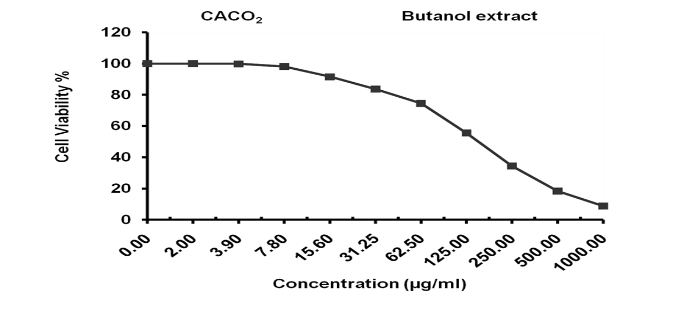** |
| **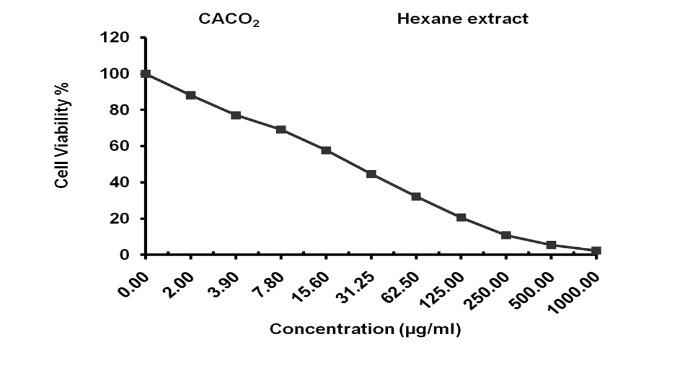** | **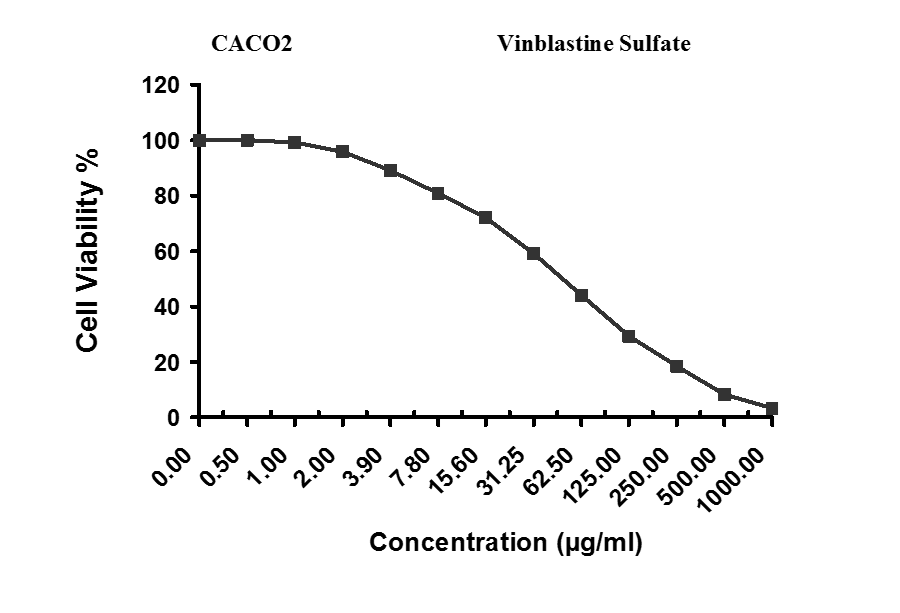** |
| **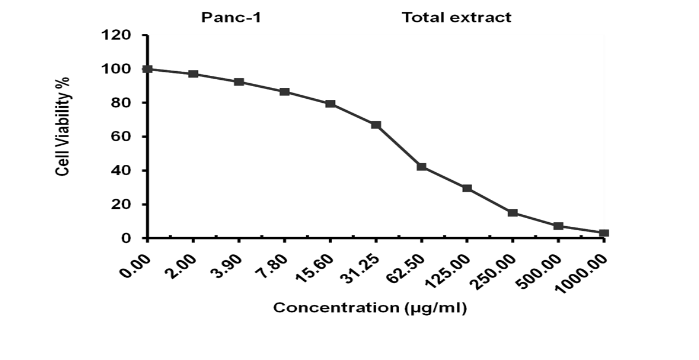** | **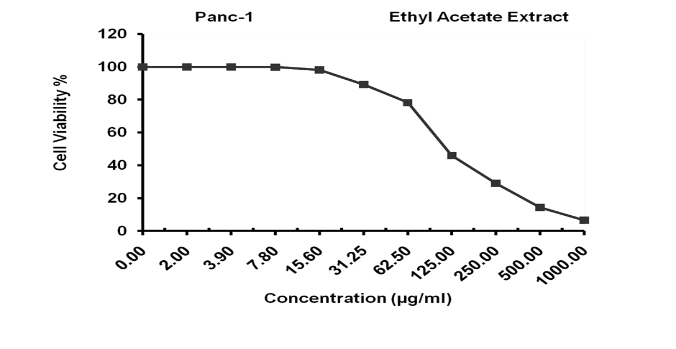** |
| **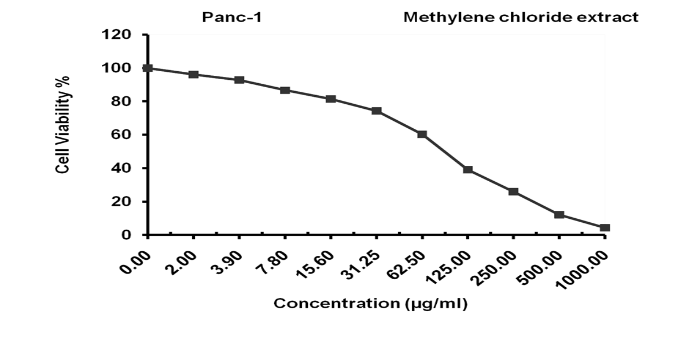** | **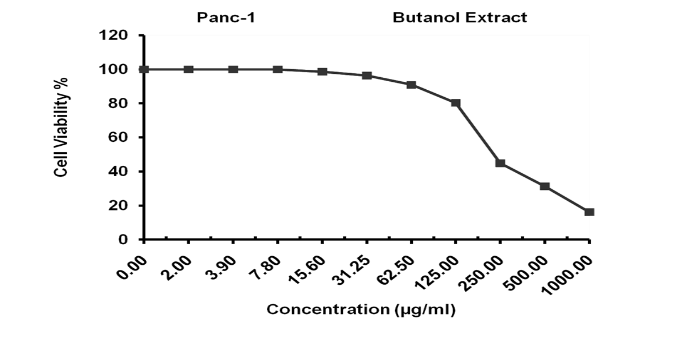** |
| **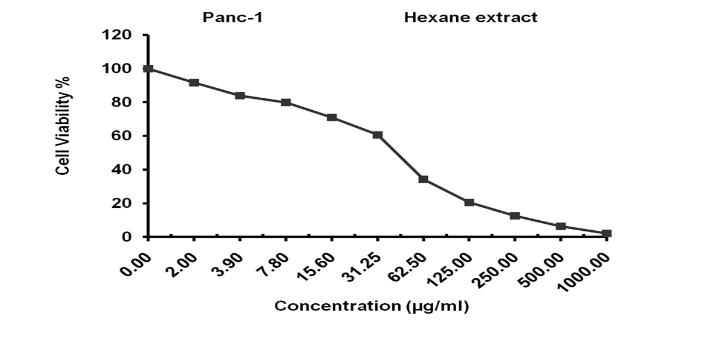** | **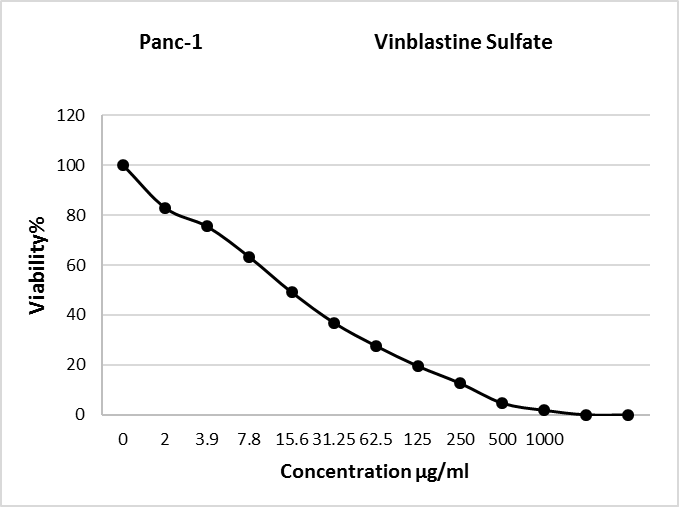** |
| **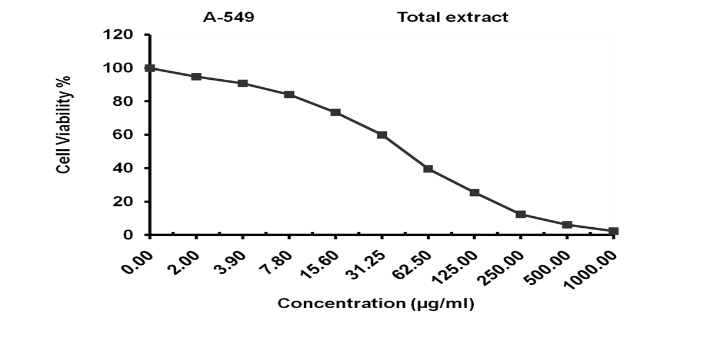** | **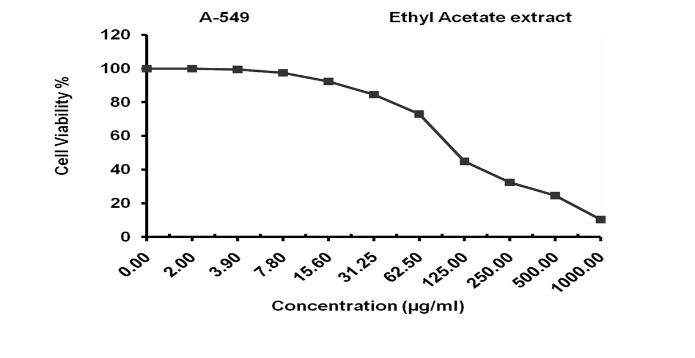** |
| **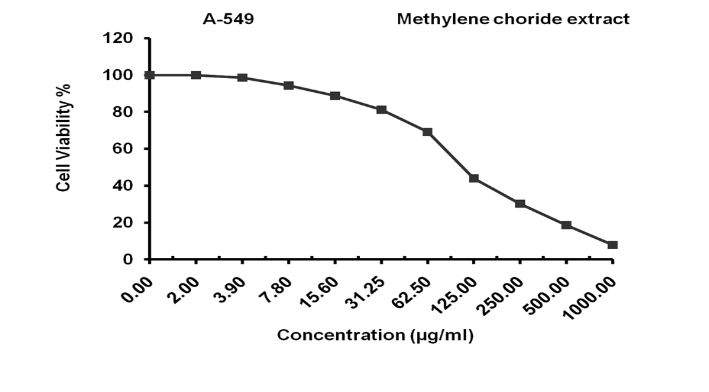** | **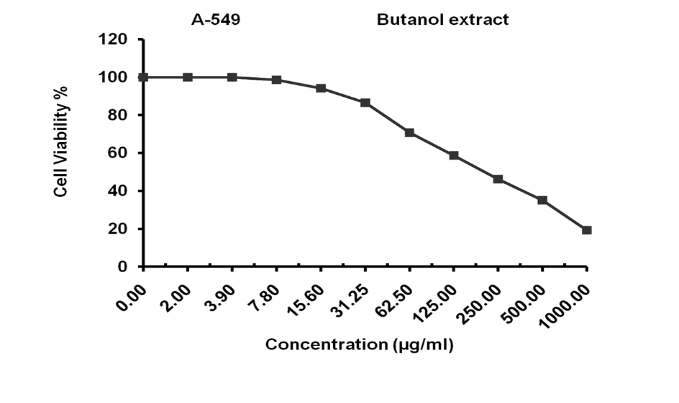** |
| 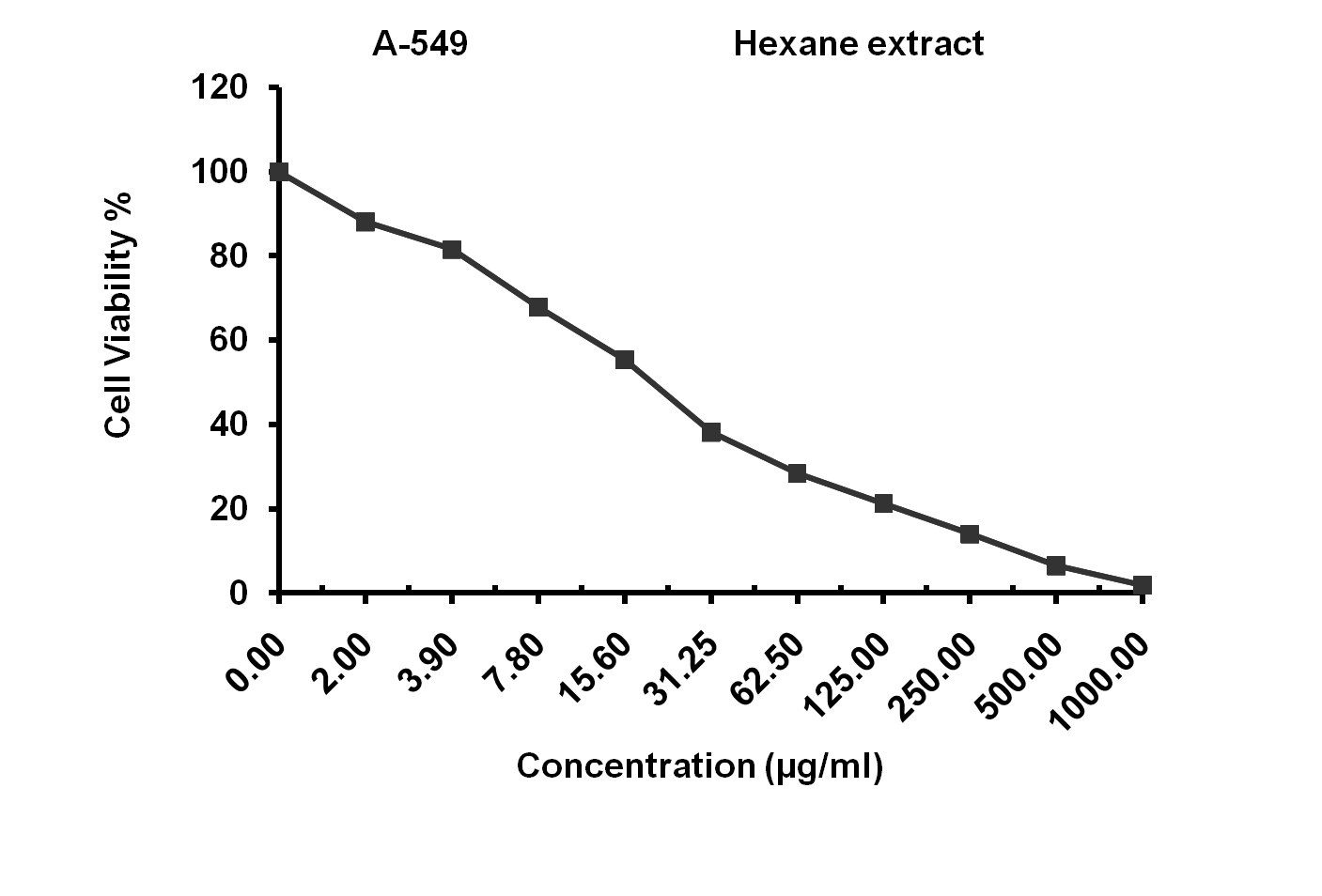 | **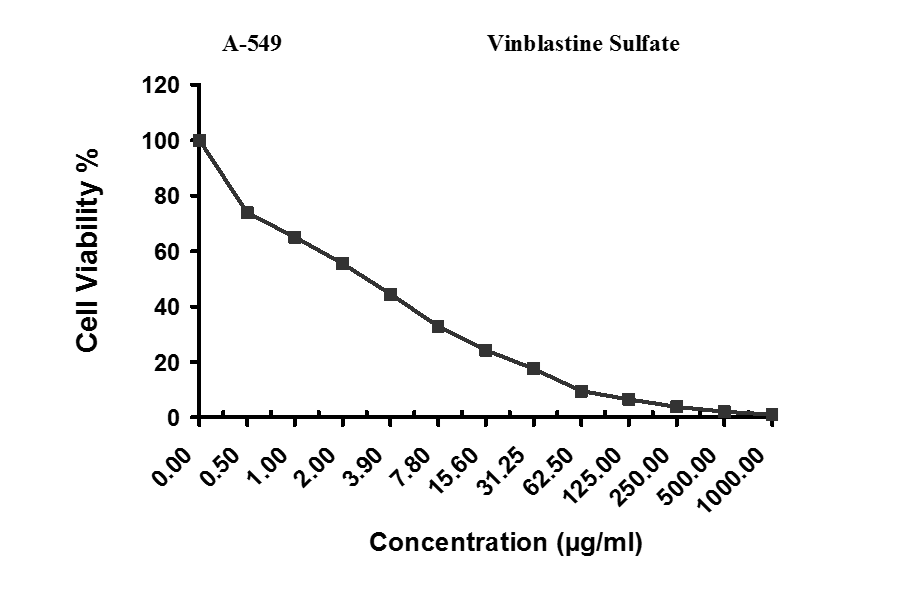** |
| **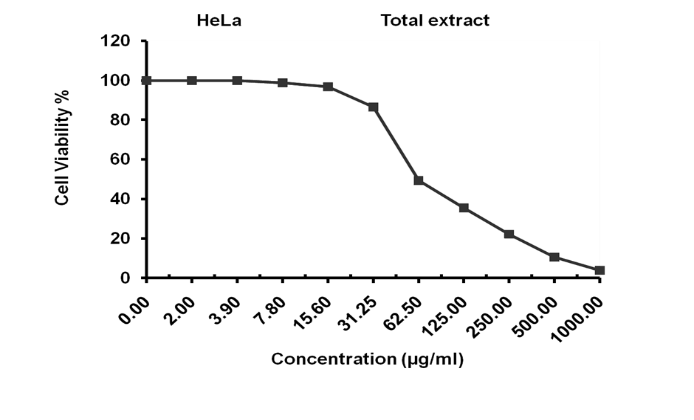** | **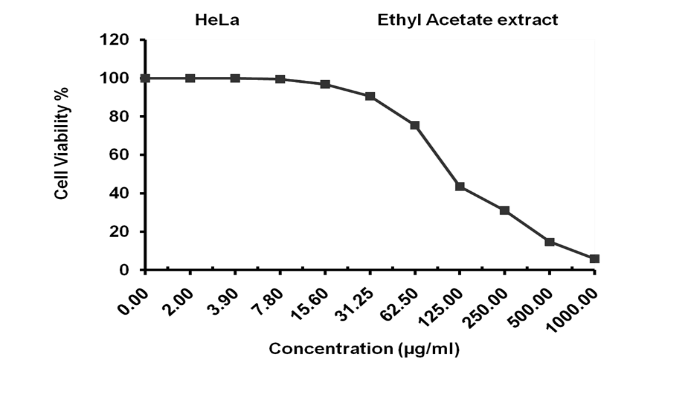** |
| **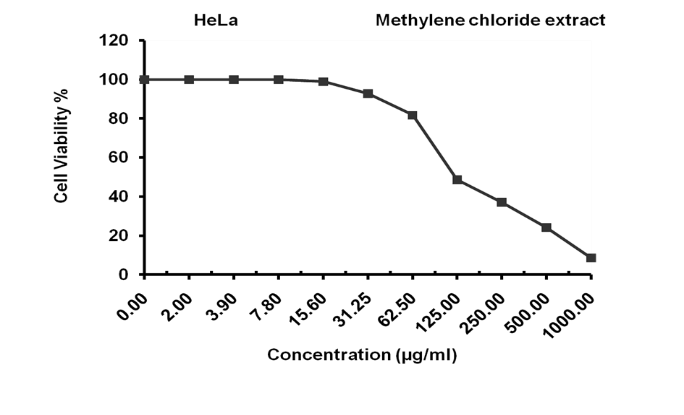** | **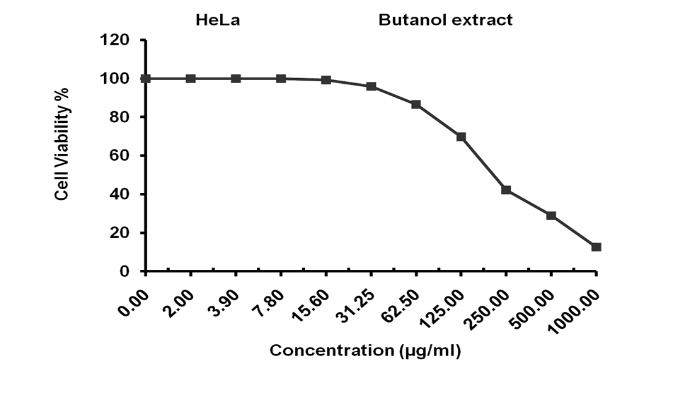** |
| **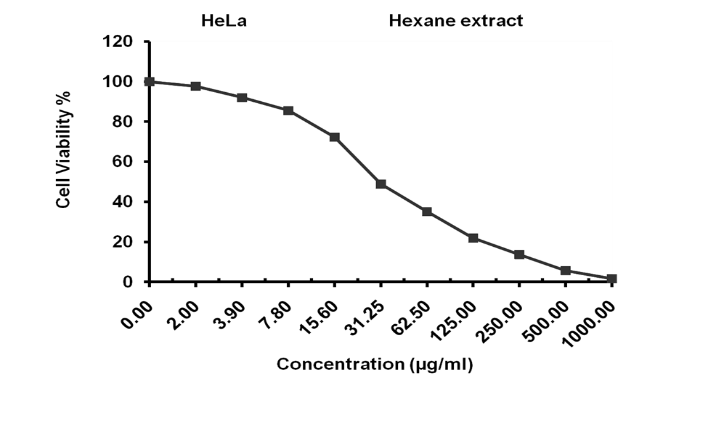** | **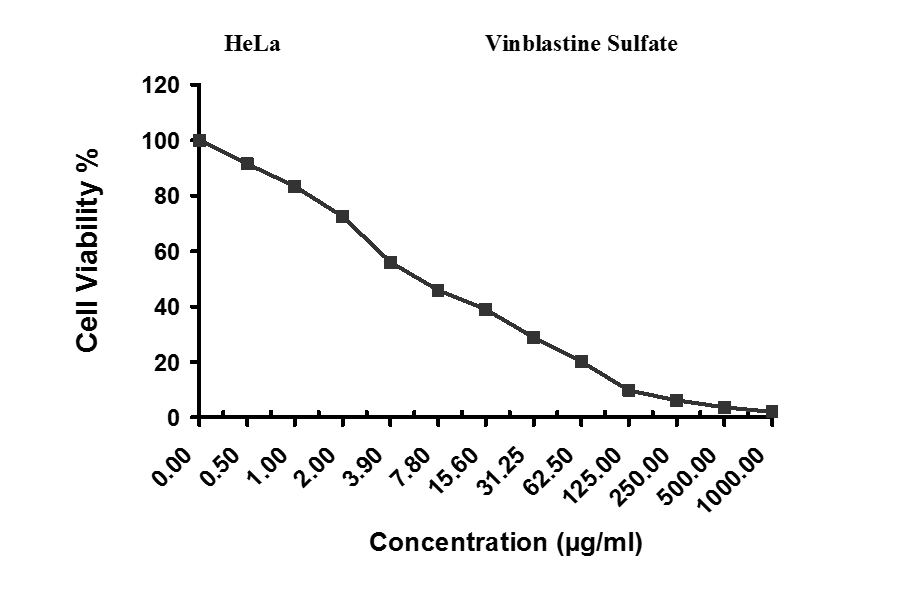** |

**Figure S1:** Comprehensive dose-response curves showing the cytotoxic effects of *Moringa oleifera* crude extract and its various fractions against cancer cell lines.


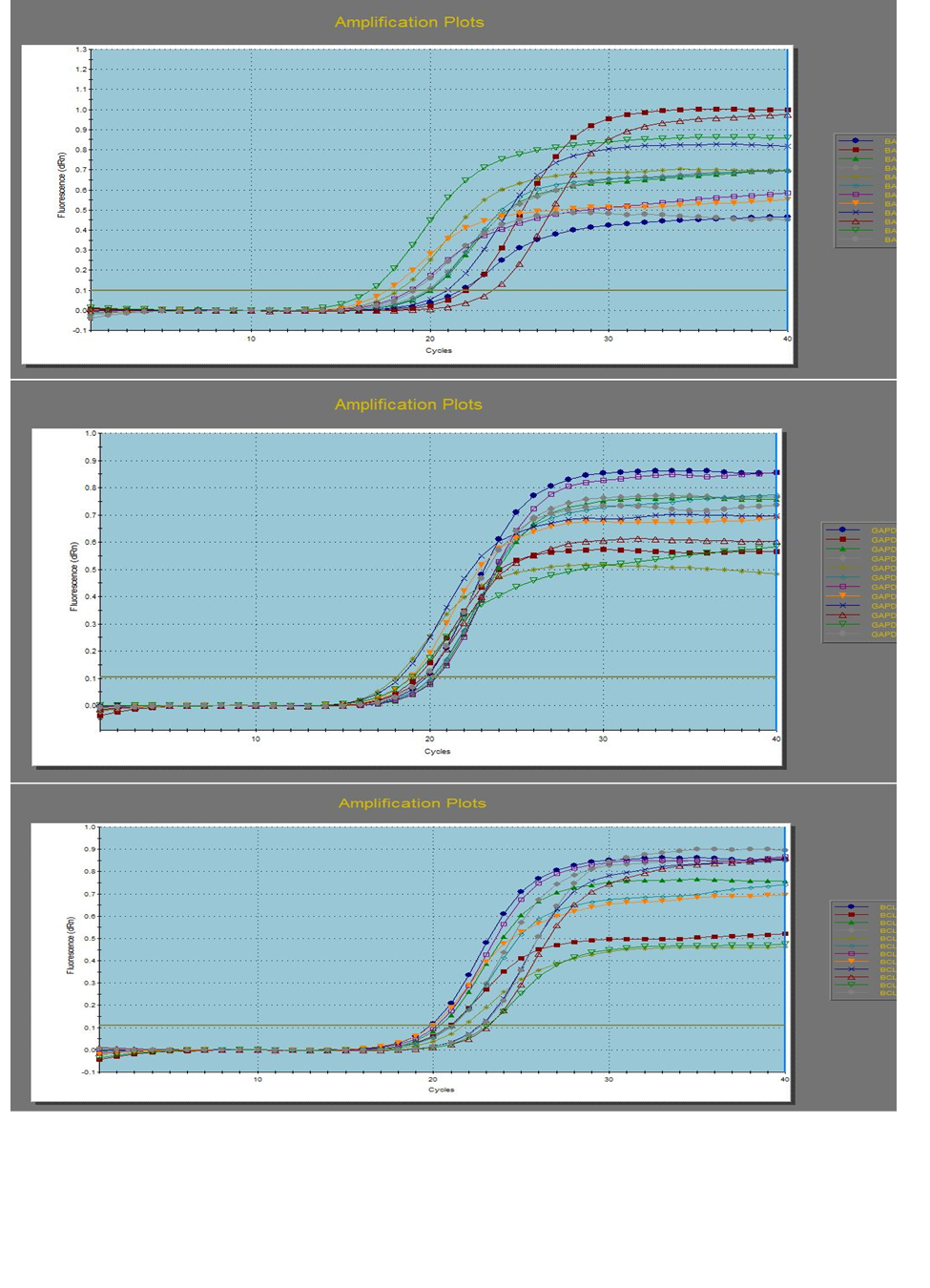


**Figure S2:** Representative qPCR Amplification Profiles and Melting Curve Analysis for target and reference Genes (GAPDH, BAX, and BCL-2).


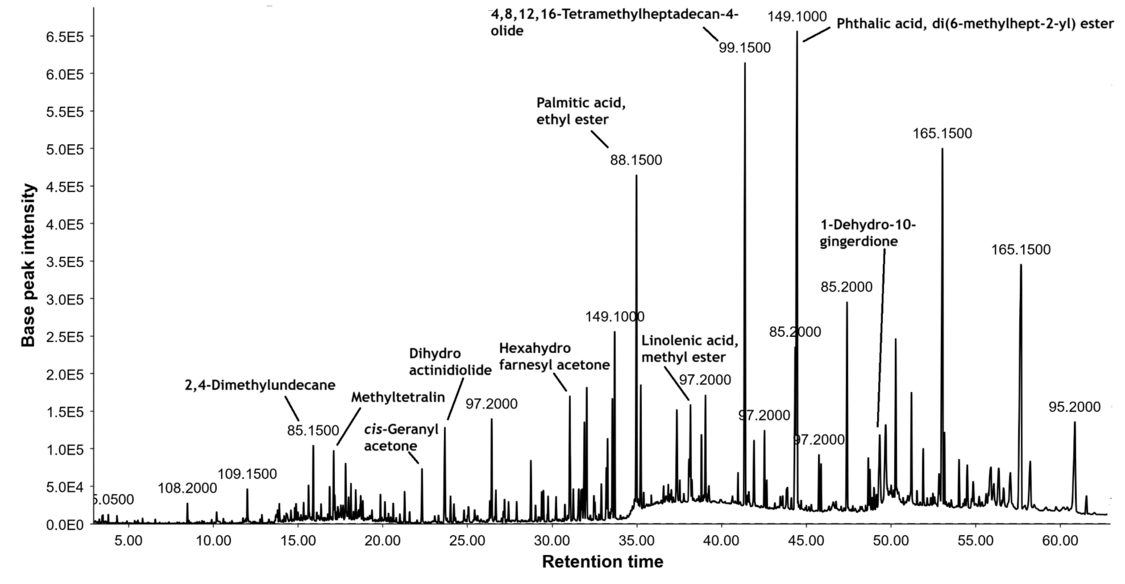


**Figure S3.** GC-MS chromatogram analysis of the hexane fraction of *Moringa oleifera*.
